# Sphingolipid-mediated vesiculation in multidrug-resistant *Sphingobacterium detergens* under polymyxin B stress

**DOI:** 10.1128/aem.01726-25

**Published:** 2025-12-03

**Authors:** Jihyeon Min, Yewon Woo, Yerim Park, Woojun Park

**Affiliations:** 1 Laboratory of Molecular Environmental Microbiology, Department of Environmental Science and Ecological Engineering, Korea University 218725, Seoul, Republic of Korea; Centers for Disease Control and Prevention, Atlanta, Georgia, USA

**Keywords:** multi-drug resistance, polymyxin, lipid raft, microdomain, outer membrane vesicle, cationic peptide

## Abstract

Among environmentally widespread *Sphingobacterium* species, the animal feces isolate *Sphingobacterium detergens* E70 exhibited multidrug antibiotic resistance despite carrying few annotated antibiotic resistance genes (ARGs), prompting investigation of non-canonical mechanisms. This observation aligns with comparative genomic analysis of 62 *Sphingobacterium* genomes, which revealed only two to four ARGs per strain, typically associated with macrolide, phenicol, or tetracycline resistance. Remarkably, strain E70 lacked canonical ARGs yet displayed high-level resistance across nine antibiotics, including β-lactams and polymyxins. Scanning and transmission electron microscopy identified polymyxin B (PMB) as the most potent envelope-disrupting agent, revealing extensive outer-membrane vesiculation following PMB exposure. Fluorescence-based cytometric and microscopic analyses revealed PMB-induced phenotypic alterations, characterized by enhanced biofilm formation, modified surface charge, and changes in lipid composition. Inhibition of sphingolipid biosynthesis with myriocin markedly reduced vesiculation and impaired growth, indicating that sphingolipids contribute to envelope integrity and stress adaptation under PMB treatment. Confocal imaging with dansyl-labeled PMB showed that myriocin-mediated sphingolipid depletion made the membrane more permeable to PMB and reduced surface fluorescence, reflecting altered membrane environments. Together, these findings uncover a previously unrecognized role of sphingolipids in PMB resistance, that is, by promoting outer membrane vesicle-mediated membrane remodeling, sphingolipids enhance PMB resilience in *S. detergens* E70 and potentially in other sphingolipid-producing bacteria.

**IMPORTANCE:**

Environmental bacteria often display antibiotic tolerance without carrying canonical resistance genes. We show that *Sphingobacterium* exploits sphingolipid-dependent outer membrane vesiculation as a structural defense against polymyxin B. Blocking sphingolipid biosynthesis with myriocin suppressed vesiculation and sensitized cells to polymyxin B, indicating that these rare bacterial lipids provide essential sites for drug interaction and membrane remodeling. Our findings reveal a lipid-driven mechanism of vesiculation and highlight sphingolipid metabolism as a potential therapeutic target.

## INTRODUCTION

The rising prevalence of multidrug-resistant (MDR) Bacteroidota members such as *Sphingobacterium* and *Chryseobacterium* in environmental reservoirs has become a pressing public health concern linked to clinical settings (1). Unlike clinical MDR strains, which often acquire resistance through mobile genetic elements (MGEs), environmental bacteria frequently exhibit antibiotic resistance phenotypes despite carrying only a limited repertoire of known antibiotic resistance genes (ARGs) (2). The high resistance in MDR bacteria may be partially reinforced by the formation of biofilms and the production of extracellular polymeric substances, both of which can contribute to increased tolerance by providing physical protection against antimicrobial stress (3). Mutations in antibiotic target sites are another factor that may contribute to antimicrobial tolerance (4). Structural modifications of penicillin-binding protein 3 in *Escherichia coli*, such as YRIN or YRIK four-amino-acid insertions after position 333, can reduce the efficacy of β-lactam antibiotics, including aztreonam and meropenem (5). Some clinical isolates, including *Klebsiella* and *Acinetobacter*, have intrinsic tolerance to cationic antimicrobial peptides (CAMPs), further highlighting the diversity of structural and physiological adaptations (6, 7). For instance, polymyxin B (PMB), a CAMP, interacts with negatively charged lipopolysaccharide (LPS) and disrupts the outer membrane through pore formation (8, 9). However, structural modifications of lipid A, such as the addition of phosphoethanolamine (PEtN) by the *eptA* gene product or 4-amino-4-deoxy-L-arabinose (L-Ara4N) decoration by the *arnT* gene product, reduce the net negative charge and block CAMP binding, thereby conferring resistance even in the absence of canonical ARGs (10). Collectively, these observations underscore how envelope-associated mechanisms limit the effective activity of antibiotics by restricting drug access or sequestering antimicrobial compounds.

Members of the phylum Bacteroidota, including *Sphingobacterium*, are notable for their broad ecological distribution and pronounced intrinsic resistance, which arises from their distinctive outer membrane architecture, reduced permeability to hydrophobic compounds, and constitutive expression of efflux pumps (11). In addition, their atypical membrane composition, enriched in sphingolipids and branched-chain fatty acids, contributes to envelope rigidity and limits antibiotic penetration (12, 13). Sphingolipids are complex amphipathic lipids composed of a long-chain sphingoid base N-acylated to form ceramide, which can be further modified into sphingomyelin and diverse glycosphingolipids (14). In eukaryotes, sphingolipids, including ceramides and sphingomyelins, are abundant and functionally central, forming a major component of the outer leaflet of the plasma membrane, where they organize ordered membrane microdomains that mediate signaling and receptor function (15, 16). In contrast, sphingolipids occur only sporadically in prokaryotes and are largely confined to specific lineages, such as members of the phylum Bacteroidota and certain Alphaproteobacteria (17, 18). Additionally, in *Bacteroides*, sphingolipids within membrane microdomains have been proposed to initiate signaling cascades, thereby facilitating stress responses and promoting bacterial persistence in the intestinal environment (12). Unlike eukaryotic cells, bacterial sphingolipid synthesis is generally limited to simple forms such as ceramide or dihydroceramide, largely because of the absence of the complex glycosyltransferases and desaturases required to generate higher-order sphingolipids (19). In *Bacteroides thetaiotaomicron*, sphingolipid-enriched outer membrane vesicles (OMVs) determine vesicle biophysical properties, cargo selectivity, and host interaction dynamics (20, 21). Lipidomic profiling revealed that ceramide, ethanolamine phosphoceramide, and dihydroceramide constitute predominant sphingolipid species in OMVs, highlighting their importance in membrane architecture and implying that sphingolipid composition could influence cargo selection and vesicle biogenesis (21). Cargo selectivity, as demonstrated by comparative proteomic analyses across different culture conditions, indicated the preferential incorporation of distinct proteins into OMVs depending on the glycan present in the growth medium (22, 23).

Interestingly, sphingolipid-enriched OMVs from bacteria can integrate into host epithelial membranes and destabilize lipid raft domains, leading to tight junction disassembly and enhanced permeability while simultaneously delivering immunomodulatory molecules that shape host–pathogen interactions (24). In *B. thetaiotaomicron*, up to 24% of the total lipid content consists of ceramide (d18:1/24:1), further emphasizing the abundance of sphingolipids that may contribute to OMV–host membrane interactions (25). However, whether sphingolipids directly modulate antibiotic responses has not been established. In this study, we investigated OMV production by *Sphingobacterium detergens* E70 under PMB stress using scanning electron microscopy (SEM), transmission electron microscopy (TEM), and confocal laser scanning microscopy (CLSM). To test the role of sphingolipids, we employed myriocin, an inhibitor of sphingolipid biosynthesis, and observed suppressed vesiculation across multiple sphingolipid-producing bacterial species under PMB exposure. Myriocin inhibits serine palmitoyltransferase (SPT), the first committed enzyme in *de novo* sphingolipid biosynthesis (26). Collectively, our results demonstrate that PMB-induced vesiculation depends on sphingolipid biosynthesis, providing evidence for a lipid-driven mechanism of antibiotic stress adaptation in *Sphingobacterium*.

## RESULTS

### Genomic analysis of multidrug-resistant *Sphingobacterium* species

**Fig 1:**
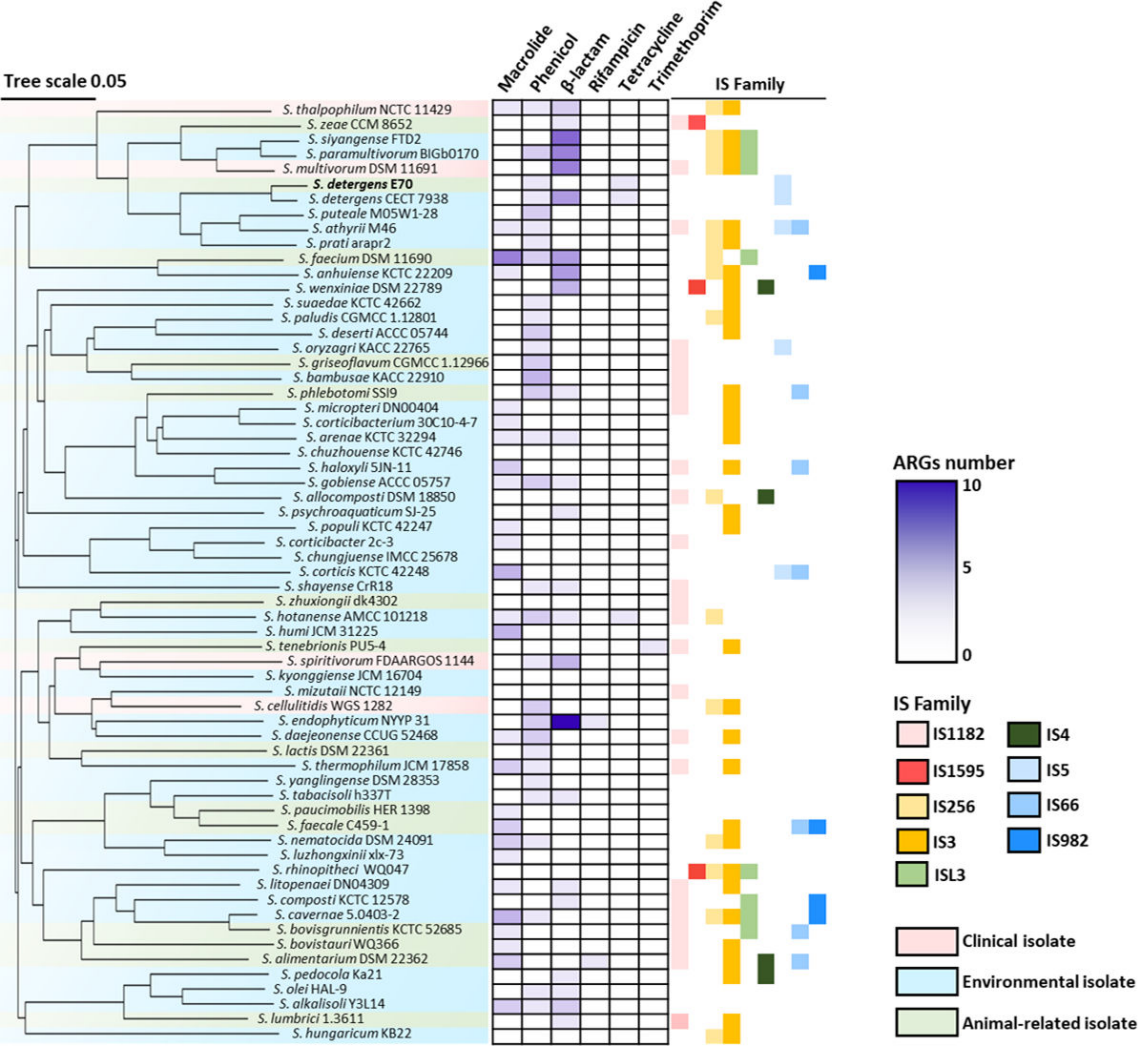
Genomic comparison and ARG prediction in the *Sphingobacterium* species. The genomes of the *Sphingobacterium* species were analyzed using CLC Genomics Workbench v22 with CARD and ResFinder databases (identity > 30%, length > 50%).

We examined the antibiotic susceptibility of the feces-derived isolate *Sphingobacterium detergens* E70 against nine agents, encompassing β-lactams, azithromycin, and polymyxin B (PMB) (Fig. S1A). Minimum inhibitory concentration (MIC) assays showed that the strain exhibited strong resistance, most notably to colistin (512 µg/mL), PMB (256 µg/mL), and azithromycin (256 µg/mL). Because the genus *Sphingobacterium* synthesizes endogenous sphingolipids that are incorporated into the outer membrane and can influence membrane organization and permeability, we transiently suppressed sphingolipid synthesis using a sub-inhibitory concentration of myriocin (5 µM). Myriocin targets serine palmitoyltransferase (SPT), the rate-limiting enzyme initiating *de novo* sphingolipid synthesis, through pyridoxal-5′-phosphate-mediated adduct formation followed by covalent modification of the catalytic lysine by a C18 aldehyde, resulting in persistent enzyme inactivation (26). We then reassessed MICs for nine antibiotics in the presence of myriocin (Fig. S1B). Overall, myriocin produced little change in susceptibility; however, reduced sphingolipid abundance is expected to exert heterogeneous effects across drugs. Notably, the MIC for PMB decreased twofold, whereas that for colistin remained unchanged, although growth at 256 µg/mL was diminished (Fig. S1B). Overall, myriocin produced little change in susceptibility; however, reducing sphingolipid abundance is expected to yield heterogeneous effects across drugs. Notably, the MIC for PMB decreased twofold, whereas the MIC for colistin was unchanged, although growth at 256 µg/mL declined (Fig. S1B). At sub-inhibitory levels, myriocin is expected to reduce sphingolipids and perturb sphingolipid-rich microdomains, which may either increase lipid A exposure to polymyxin or loosen local membrane packing to facilitate acyl-tail insertion, thereby enhancing binding, accumulation, and susceptibility (19, 27). In addition, myriocin appeared to increase susceptibility to meropenem and doxycycline, potentially via enhanced OmpA-like channel-mediated entry or slight loosening of outer membrane organization, which could allow easier diffusion of these relatively small agents (Fig. S1B) (28, 29). Consequently, sphingolipid modulation may remodel the outer membrane landscape in a manner that differentially alters antibiotic penetration pathways. Given the elevated MICs despite few annotated ARGs, we focused on resistance and tolerance mechanisms. To explore the genetic basis of resistance, *in silico* prediction of ARGs was performed using CLC Genomics Workbench with the CARD and ResFinder databases (minimum nucleotide identity >30%; minimum length >50%). Only canonical ARGs identified by genomic annotation were analyzed. Broad-spectrum efflux systems, including TolC-dependent and resistance-nodulation-division (RND)-type transporters, were excluded because they participate in diverse envelope-associated processes, notably in stress responses to detergents, bile salts, and oxidative damage (30, 31). Surprisingly, only two ARGs were detected in the E70 strain: *catA2*, encoding chloramphenicol acetyltransferase, and *tetX*, a flavin-dependent monooxygenase conferring tetracycline resistance (Fig. 1). No β-lactamase or aminoglycoside resistance genes were identified, revealing a striking mismatch between genotype and phenotype. Similarly, applying lower identity thresholds (>20%) did not lead to the detection of additional ARGs. Comparative genomic analysis was subsequently performed across 62 publicly available *Sphingobacterium* genomes using Mashtree. The data set encompassed isolates from diverse habitats, including soil, freshwater, plant rhizospheres, insect guts, and animal feces, with only four strains (6.5%) originating from clinical sources (Fig. 1). Despite their varied origins, most genomes encoded only two to four ARGs, primarily belonging to the macrolide, phenicol, and tetracycline classes.

Phylogenetic analysis revealed that strain E70 clustered most closely with *S. detergens* CECT 7938, a soil isolate (32). Although the core genomic architecture was conserved between the two strains (genome size, 5.9–6.7 Mbp; GC content, 39.9–43.6%), the CECT 7938 genome carried additional resistance determinants, including two β-lactamase variants (*blaVEB-4*, *blaVEB-5*), *catA2*, and *tetX6* (Fig. 1). At the genus level, most *Sphingobacterium* genomes contained only a few ARGs and a limited number of insertion sequence (IS) families, such as IS1182, IS256, and IS3, suggesting relatively infrequent horizontal gene transfer (HGT) (33). IS3 was the most prevalent element, present in 50.8% of genomes, whereas IS1595 was rare, occurring in 4.8% of genomes. The consistent presence of only a small set of ARGs and IS families indicates long-term conservation of the *Sphingobacterium* genomic backbone, with limited HGT shaping the resistome (34, 35). Consequently, these observations suggest that the resistance phenotype of *Sphingobacterium* is more likely driven by intrinsic envelope properties or unknown cellular mechanisms rather than by frequent horizontal acquisition of resistance genes (36, 37). For comparison, phylogenetic analyses of *Acinetobacter* revealed shorter branch lengths and denser clustering, reflecting more frequent genetic exchange and higher HGT rates (Fig. S2). This pattern is consistent with the natural competence and IS abundance of *Acinetobacter* (approximately 250 elements across 15 IS families) (38). Similarly, a survey of 13 Gammaproteobacterial species, including *Acinetobacter* and *Klebsiella*, revealed an average of eight ARGs and 59 IS elements—far exceeding the levels observed in *Sphingobacterium* (Fig. S3). Collectively, these findings highlight a clear discrepancy between the high levels of antibiotic resistance observed in *Sphingobacterium* and the limited number of identifiable ARGs, suggesting that intrinsic mechanisms—potentially involving sphingolipid-rich membranes—underpin their antibiotic tolerance.

### PMB-induced membrane perturbation and vesiculation

**Fig 2:**
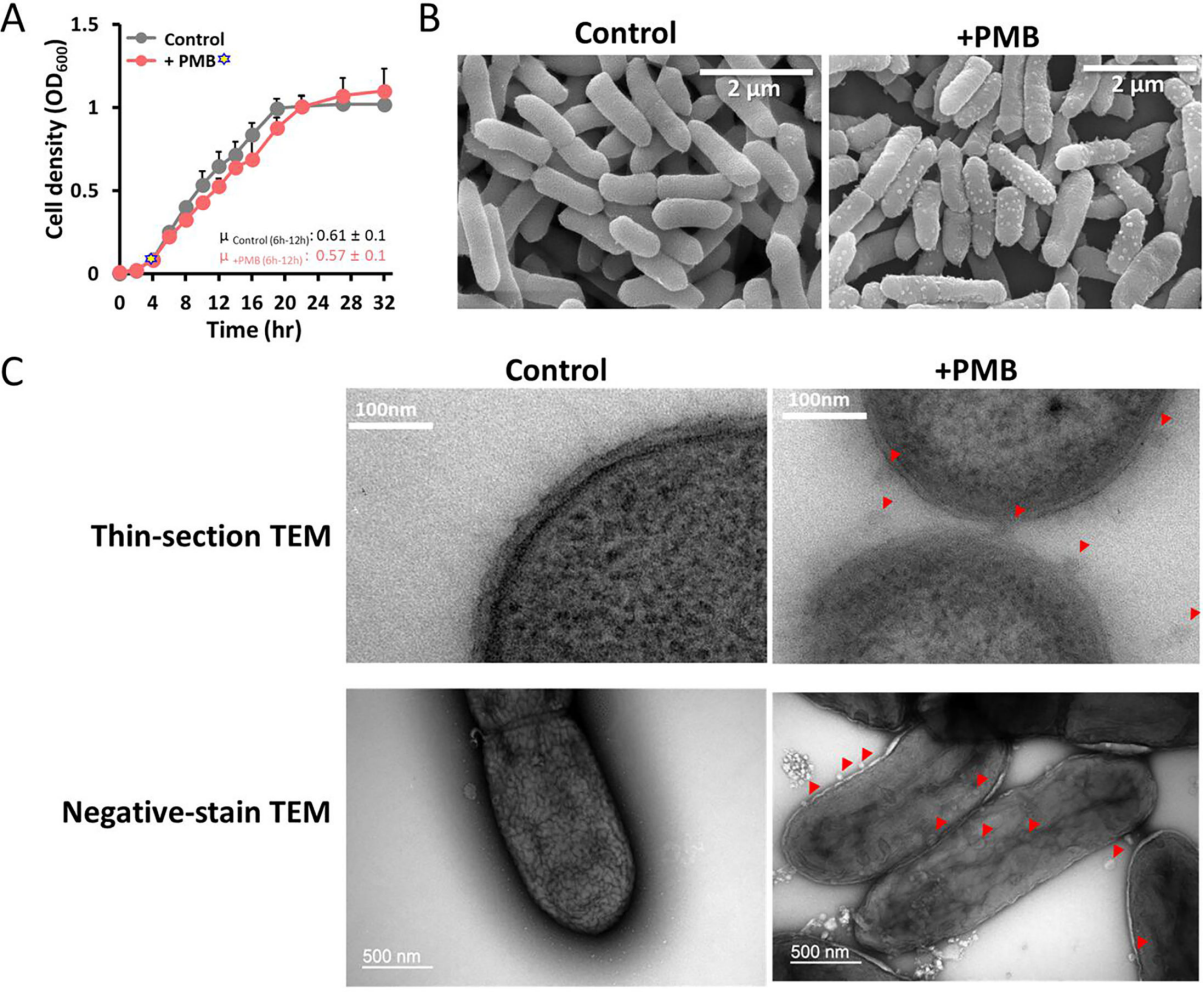
PMB-induced OMV formation and membrane remodeling in the E70 strain. (**A**) Growth curves of E70 cultured in R2A medium in the absence (black) or presence (red) of PMB at 1/4 MIC (64 µg/mL). (**B**) SEM images of E70 cells after 3 h with or without 1/4 MIC PMB (64 µg/mL). (**C**) TEM analysis of ultrathin sections and negatively stained whole cells. PMB-treated cells exhibited disorganized and flattened outer membranes, with budding vesicle-like structures at the surface (red arrowheads), indicating membrane destabilization and OMV formation. All experiments were performed in three independent replicates.

The disparity between the limited ARG content and the high antibiotic tolerance of the E70 strain suggests that intrinsic envelope-associated features may contribute to resistance, including peptidoglycan remodeling, membrane lipid modification, efflux activity, or protective OMV production (7, 29, 33). To examine antibiotic-induced surface alterations, SEM was performed following exposure to the three antibiotics with the highest MIC values and two additional antibiotics with distinct modes of action: PMB, colistin, azithromycin, meropenem, and oxytetracycline (Figure 2; Fig. S1 and S4). Each antibiotic was tested at one-quarter MIC for 3 h, corresponding approximately to the doubling time (156 min) of the E70 strain (Fig. 2; Fig. S4). Cationic LPS-targeting peptides, such as PMB and colistin, disrupted outer membrane packing, inducing surface blebbing and OMV formation (Fig. 2; Fig. S4). Despite inhibiting peptidoglycan cross-linking, meropenem caused neither elongation nor lysis, whereas azithromycin and oxytetracycline produced no discernible envelope remodeling under short sub-MIC exposure (Fig. S4). In clinical contexts, meropenem exhibits broad-spectrum activity by covalently acylating the active sites of PBP2 and L,D-transpeptidases, thereby preventing peptidoglycan cross-linking and weakening the cell wall without immediate lysis (31). The lack of envelope remodeling under short sub-MIC exposure to azithromycin or oxytetracycline is consistent with their established modes of action on the 50S exit tunnel and the 30S A-site, respectively (39, 40). Accordingly, we focused subsequent analyses on PMB, which robustly induces outer membrane remodeling and vesiculation (Fig. 2). Growth rates remained unaffected at one-quarter MIC (64 µg/mL) PMB (μ = 0.57 ± 0.1 h⁻¹) compared with the control (μ = 0.61 ± 0.1 h⁻¹), indicating that vesiculation was not directly coupled to growth inhibition (Fig. 2A and B). TEM further supported PMB-induced membrane perturbation. Thin-section TEM revealed blurred, disorganized outer membranes and vesicle budding (Fig. 2C, red arrowheads), while negative-stain TEM confirmed flattened and disrupted surfaces with clustered vesicle protrusions adhering to the envelope (Fig. 2C). Together, SEM and TEM analyses demonstrate that CAMPs directly perturb the outer membrane and induce vesiculation.

To assess whether PMB-induced vesiculation was medium-dependent, we examined OMV formation in LB (μ = 0.89 ± 0.1 h⁻¹) and BHI (μ = 1.17 ± 0.2 h⁻¹) media, both of which supported higher growth rates than R2A medium (Fig. S5A). The one-quarter MIC of PMB increased to 512 µg/mL in both nutrient-rich media, consistent with enhanced tolerance. However, SEM analysis revealed no detectable OMV formation in LB or BHI, indicating that nutrient composition influences the membrane response to PMB (Fig. S5B). These observations suggest that the composition of R2A medium strongly promotes PMB-induced vesiculation, and identifying the specific contributing factors will require further study using defined minimal media (41). To further determine whether sphingolipid biosynthesis responds to PMB in a medium-dependent manner, we analyzed the expression of *spt*, which encodes serine palmitoyltransferase (SPT)—the first committed enzyme in the sphingolipid biosynthetic pathway (Fig. S6A). Expression was monitored during the early exponential phase (up to 120 min) based on the doubling time (156 min), as stress-responsive lipid metabolism genes often show delayed induction (42). In R2A medium treated with one-quarter MIC PMB (64 µg/mL), *spt* expression increased steadily, reaching a 3.98-fold upregulation at 120 min compared with the control (Fig. S6B). By contrast, expression was markedly downregulated in LB (0.54-fold) and BHI (0.88-fold) under identical conditions (Fig. S6C). Cross-medium comparison at 120 min further confirmed reduced *spt* expression in LB and BHI relative to R2A (Fig. S6D). Stress-induced upregulation of *spt*, encoding a sphingolipid synthase that interfaces with fatty acid metabolism, has also been reported in other organisms, such as yeast and *Caulobacter*, where genes associated with fatty acid synthesis are commonly upregulated more than twofold under oxidative stress conditions (43, 44). Collectively, these findings indicate that PMB-induced vesiculation in the E70 strain is closely linked to sphingolipid biosynthesis and that nutrient composition modulates both transcriptional activation and membrane remodeling.

### Remodeling of the membrane region under PMB stress

**Fig 3:**
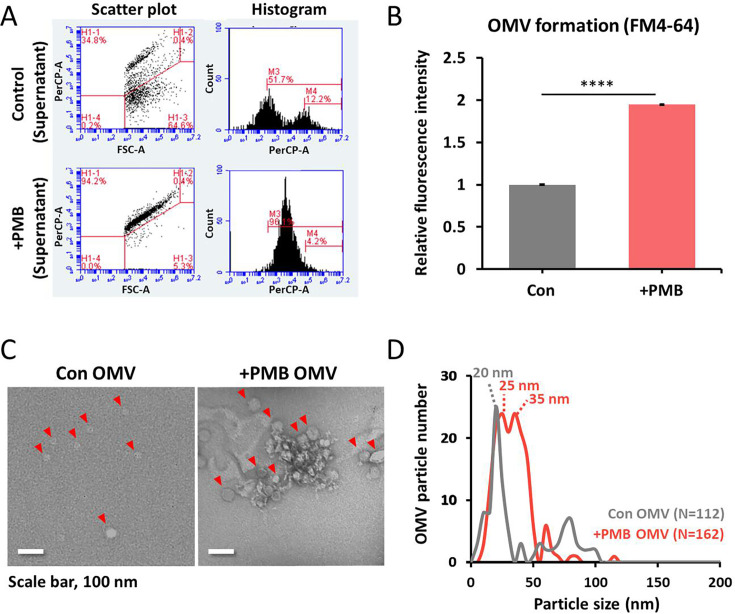
Hyper-vesiculation of the E70 strain under the PMB treatment condition. (**A**) OMV production was assessed by flow cytometry using FM4-64 staining of cell-free supernatants. (**B**) MV quantification was performed by FM4-64 staining of cell-free culture supernatants. (**C**) TEM analysis of purified OMV showed well-dispersed vesicles in the control sample and aggregated or fused OMV clusters in the PMB-treated sample. (**D**) Nanoparticle tracking analysis (NTA) of isolated OMVs under each condition. (*, *P* < 0.5; **, *P* < 0.01; ***, *P* < 0.005; ****, *P* < 0.001; NS, not significant).

To characterize vesicles produced under PMB stress, cell-free supernatants from control and PMB-treated cultures were stained with the lipophilic dye FM4-64 and analyzed by flow cytometry (Fig. 3A). A distinct population of fluorescent particles (94.25%) with a narrow and symmetric size distribution was detected under PMB treatment, consistent with homogeneous OMV production. Furthermore, FM4-64–based fluorescence analysis showed a 1.9-fold increase under PMB treatment, consistent with enhanced OMV release in culture supernatants (Fig. 3B). Purification by OptiPrep density gradient and visualization by negative-stain TEM revealed that control OMVs appeared as well-dispersed individual structures, whereas PMB-induced OMVs tended to aggregate or fuse into clusters (Fig. 3C). Nanoparticle tracking analysis further showed that control OMVs had a dominant size peak at 20 nm, while PMB-induced OMVs displayed two peaks at 25–35 nm, indicating larger vesicles under antibiotic stress (Fig. 3D). The increased vesicle size may reflect altered lipid composition or membrane curvature under PMB stress. Both vesicle populations were smaller than the typical size range (40–200 nm) reported for Gram-negative bacteria (45, 46). In addition, no detectable protein bands were observed by SDS–PAGE in either condition, with only smear-like patterns at high protein loading (100 µg/mL), suggesting a high lipid-to-protein ratio (Fig. S7) (47). The detection limit of Coomassie staining was approximately 8–16 ng of total protein per lane, indicating that OMV protein content was below the detectable threshold (48). SEM analysis further showed that OMVs became evident on the cell surface before completion of a single doubling time (Fig. S8), indicating that vesiculation in the E70 strain represents a rapid, physical response to PMB exposure. The rapid production of OMVs with minimal protein cargo suggests that PMB-induced vesiculation in the E70 strain occurs primarily through physical membrane remodeling rather than *de novo* macromolecular synthesis and that these OMVs are structurally distinct from protein-enriched vesicles described in other Gram-negative bacteria (6, 22, 23).

**Fig 4:**
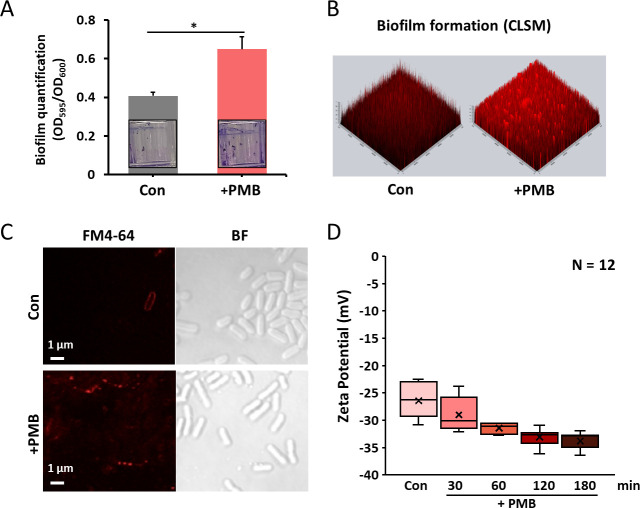
Biofilm formation and membrane surface properties were altered in the E70 strain following PMB treatment. (**A**) Biofilm biomass was quantified after 24 h incubation with or without PMB (32 µg/mL) using crystal violet staining. Representative test tube images showing stained biofilms under each condition. These experiments were performed in three independent replicates. (**B**) Biofilm formation was visualized by CLSM. PMB-treated samples exhibited thicker and denser biofilms compared to the control. (**C**) Membrane staining using FM4-64 was performed to visualize surface lipid distribution. CLSM images revealed increased membrane-associated fluorescence under PMB, indicating lipid accumulation or vesicle adhesion on the cell surface. (**D**) The zeta potential of E70 cells was measured after 180 min of PMB exposure. PMB-treated cells exhibited a significantly more negative surface charge compared to the control condition. (*, *P* < 0.5; **, *P* < 0.01; ***, *P* < 0.005; ****, *P* < 0.001; NS, not significant).

To investigate membrane-associated phenotypes under PMB stress, biofilm formation and surface charge were examined. Crystal violet staining revealed a 1.5-fold increase in biofilm production under PMB treatment, a finding confirmed by CLSM, which showed denser biofilm architecture within 24 h (Fig. 4A and B). To assess direct drug–membrane interactions, fluorescence imaging was performed using dansyl-labeled PMB (dansyl-PMB [2 µg/mL, corresponding to the MIC for *A. baumannii*]) (Fig. S9). At this concentration, *A. baumannii* cells exhibited strong dansyl-PMB fluorescence, consistent with their PMB sensitivity (Fig. S9). In contrast, *S. detergens* displayed markedly weaker fluorescence, likely because 2 µg/mL is far below its inhibitory concentration (MIC = 256 µg/mL) and therefore insufficient to promote stable binding to the cell surface. At low concentrations, the weak binding of dansyl-PMB to the *S. detergens* surface likely reflects differences in outer membrane organization rather than simple charge limitation, suggesting a distinct interaction mechanism compared with PMB-sensitive bacteria. FM4-64 staining was also less efficient in *S. detergens* cells and improved only in the presence of PMB (64 µg/mL; one-quarter MIC) (Fig. S4C and S9). Together, these observations indicate that the sphingolipid-enriched outer membrane of *S. detergens* is less accessible to PMB and the lipophilic dye FM4-64 than that of typical Gram-negative bacteria, likely due to the presence of sphingolipids that restrict membrane permeability. In *A. baumannii*, PMB resistance is typically mediated by lipid A modification via PEtN transferase. The E70 genome encodes a single *eptA* homolog (40.8% identity to *E. coli*), but RT-PCR analysis revealed no change in expression under PMB stress (Fig. S6E). In addition, the E70 strain lacks the two-component regulatory system *pmrAB*, which normally controls lipid A modifications conferring resistance to CAMPs. The strong binding of dansyl-PMB despite high tolerance indicates that resistance in the E70 strain does not result from lipid A modification (8). Consistently, the zeta potential shifted from –26.3 ± 3.4 mV to –34.9 ± 1.4 mV after 180 min of PMB exposure, indicating an increase in surface negative charge associated with vesiculation (Fig. 4D). This shift is consistent with PMB disrupting the outer membrane and displacing divalent cations (e.g., Mg²^+^) that normally bridge lipid headgroups, thereby exposing more negatively charged surfaces. To assess potential changes in protein distribution, subcellular fractionation followed by SDS-PAGE was performed (Fig. S10) (48, 49). This approach enables reliable detection of compartment-specific protein reorganization associated with membrane remodeling, as demonstrated previously (4). Protein profiles were similar in inner and outer membrane fractions between control and PMB-treated cells. In contrast, the periplasmic fraction exhibited marked differences, suggesting that PMB-induced vesiculation is associated with altered periplasmic composition rather than structural changes in membrane proteins. Collectively, these findings indicate that PMB resistance in *S. detergens* E70 involves rapid, lipid-driven vesiculation accompanied by increased surface charge and periplasmic remodeling, rather than canonical lipid A modification.

### Sphingolipid-dependent vesicle formation under PMB stress

**Fig 5:**
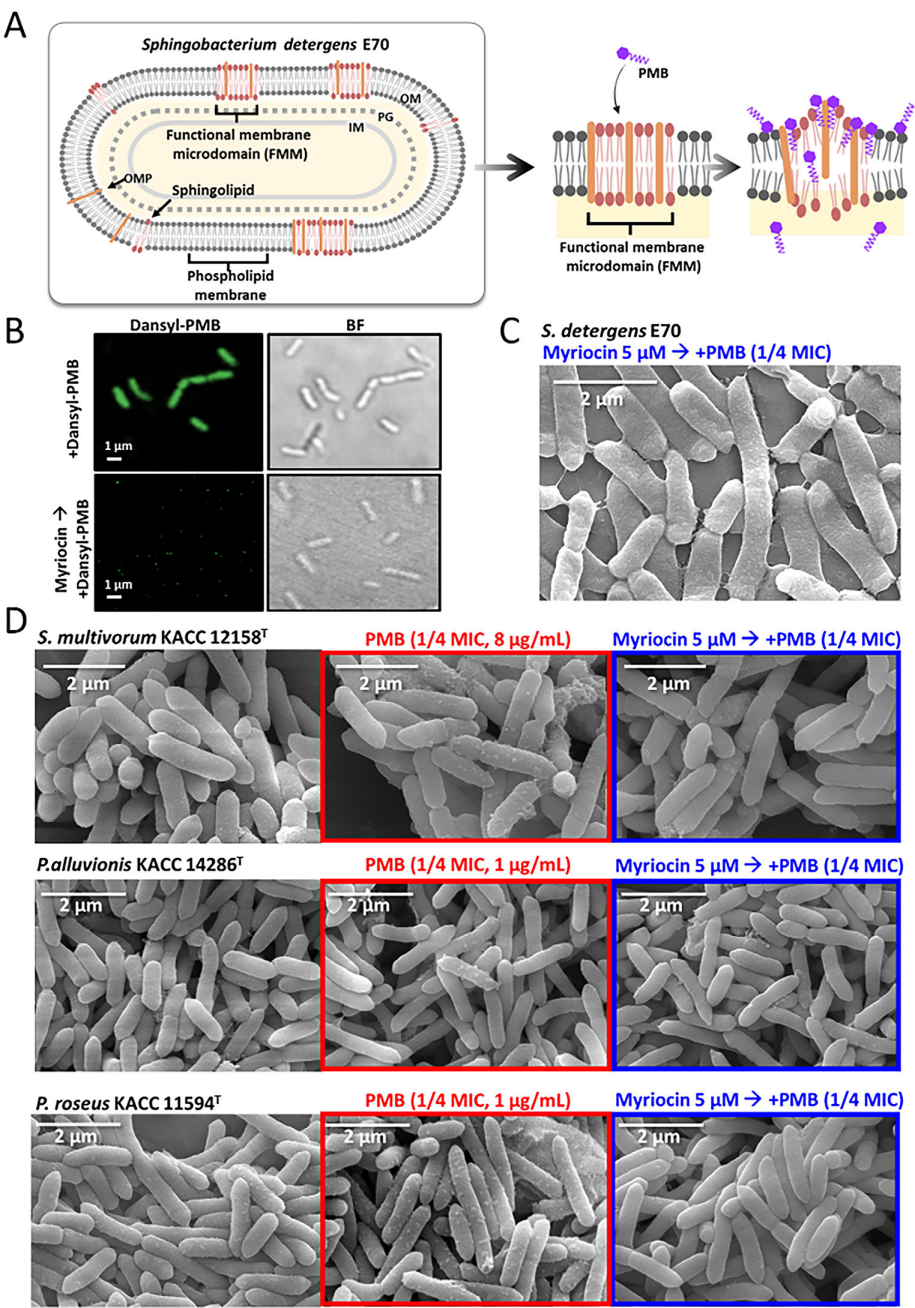
Sphingolipid-mediated modulation of membrane vesiculation under PMB stress in sphingolipid-producing bacteria. (**A**) A schematic illustration depicting the proposed mechanism by which sphingolipid-containing FMMs in *S. detergens* E70 serve as preferential binding sites for PMB. (**B**) Fluorescence imaging using dansyl-PMB was performed to assess membrane binding in the presence or absence of myriocin (5 µM), an inhibitor of sphingolipid biosynthesis. (**C**) SEM images of E70 cells treated with PMB (1/4 MIC, 64 µg/mL) with or without myriocin. (**D**) SEM analysis of three additional sphingolipid-producing species treated with PMB (1/4 MIC) with or without 5 µM myriocin.

The sphingolipid-producing bacterium *S. detergens* E70 contains endogenous sphingolipids, such as ceramide and dihydroceramide, which are typically localized within functional membrane microdomains (FMMs) (50). Because PMB preferentially associates with negatively charged lipids, sphingolipid-enriched regions containing anionic species, such as ceramide phosphoglycerate and inositol phosphoceramide, may serve as additional insertion sites (Fig. 5A) (51). To examine whether PMB alters lipid composition, fatty acid methyl ester (FAME) profiling was performed. Long-chain saturated fatty acids (e.g., C11:0, C12:0, C14:0, and C16:0) were reduced under PMB treatment, whereas unsaturated fatty acids (e.g., C14:1 ω5c and C15:1 ω6c) increased overall (Table S1). LC-MS analysis further revealed that dihydroceramide (d18:0/16:0) and ceramide (d18:1/24:1) levels increased 1.1- and 1.4-fold, respectively, under PMB stress (Fig. S11A and B). Thin-layer chromatography (TLC) also detected a 1.4-fold enrichment of the Rf 0.35 fraction (Fig. S11C), previously identified as ceramide phosphorylethanolamine in *Bacteroides* (52). These lipid shifts likely promote membrane curvature and destabilization, while the reduction in branched-chain fatty acids, typically associated with membrane fluidity, may increase rigidity and influence OMV stability (53, 54).

To determine whether these membrane changes are linked to sphingolipid biosynthesis, myriocin was used as a chemical inhibitor of serine palmitoyltransferase (SPT) (Fig. S11D). Attempts to genetically disrupt the *spt* gene in the E70 strain to assess its role in membrane composition were unsuccessful despite multiple approaches, including chemical transformation, electroporation, and conjugation, for reasons that remain unclear. Consequently, chemical inhibition with myriocin was employed as an alternative strategy (Fig. 5B). When E70 cells were co-treated with PMB and myriocin (5 µM), fluorescence from dansyl-PMB (64 µg/mL) was significantly reduced compared with the PMB-only condition, suggesting impaired PMB binding to the membrane (Fig. 5B). Myriocin-mediated inhibition of sphingolipid synthesis reduced dansyl-PMB surface binding, indicating that sphingolipids modulate membrane charge distribution and lipid packing, thereby influencing PMB accessibility (Fig. 5B). SEM further revealed that co-treatment with myriocin altered OMV morphology and substantially reduced vesiculation in the E70 strain under PMB exposure (Fig. 5C). To determine whether these effects were conserved among other sphingolipid-producing bacteria, SEM analyses were conducted on *S. multivorum* and two *Pedobacter* species (*P. alluvionis* and *P. roseus*). When treated with PMB alone at one-quarter MIC, all tested strains exhibited abundant vesicular structures on their surfaces (Fig. 5D). Consistent with the results for the E70 strain, vesiculation was markedly reduced in all three sphingolipid-producing species upon co-treatment with PMB and myriocin, as confirmed by SEM (Fig. 5D).

Furthermore, chemical inhibition of sphingolipid synthesis suppressed multiple phenotypes observed under PMB stress (Fig. S12). OMV production, which was prominent during PMB treatment, was barely detectable when sphingolipid biosynthesis was blocked, indicating a critical requirement for these lipids in vesiculation (Fig. 4C; Fig. S12A). Biofilm biomass decreased to approximately 0.5-fold under control conditions and ~0.4-fold under PMB exposure with myriocin treatment (Fig. S12B). CLSM imaging further confirmed that cells failed to incorporate FM4-64 under sphingolipid biosynthesis inhibition, reflecting reduced membrane sphingolipid content (Fig. S12C). These findings support the notion that, in *S. detergens*, PMB preferentially associates with membrane regions enriched in sphingolipids. Surface charge measurements also showed that the PMB-induced decrease in zeta potential was abolished in myriocin-treated cells, consistent with the loss of negatively charged lipid species (Fig. S12D) (55). Moreover, exponential-phase growth rates declined by ~0.7-fold (approximately a 24% reduction) when myriocin treatment preceded PMB exposure (Fig. S12E). Myriocin treatment alone did not affect bacterial viability, indicating that the reduced growth observed under combined treatment resulted from PMB exposure, supporting a role for sphingolipid biosynthesis in intrinsic PMB tolerance (Fig. S12E and F). Collectively, these results demonstrate that sphingolipids are essential for OMV production and membrane remodeling under PMB stress. Chemical inhibition of sphingolipid biosynthesis consistently reduced vesiculation, biofilm formation, lipid staining, and surface charge alterations across multiple sphingolipid-producing species, underscoring the central role of sphingolipids in lipid-driven stress adaptation.

## DISCUSSION

Originally isolated from the feces of a herbivorous animal, *S. detergens* E70 displayed a MDR phenotype despite carrying only two identifiable ARGs, neither of which was associated with β-lactam or aminoglycoside resistance (Fig. 1; Fig. S1). The high MIC values observed for multiple antibiotics (256–512 µg/mL) underscore a striking genotype–phenotype discrepancy, suggesting the involvement of unconventional mechanisms beyond canonical ARGs (17). Strain-level variation appears to contribute to this mismatch, as the closest relative, *S. detergens* CECT 7938, carries a broader resistome including multiple β-lactamases (56). However, even in clinically important taxa, such as *Salmonella enterica*, genotype–phenotype mismatches are common, with only 53% of non-typhoidal isolates showing concordance between ARG content and resistance phenotype (57). Similar inconsistencies have been documented in *Vibrio parahaemolyticus*, further emphasizing the role of intrinsic mechanisms, such as envelope remodeling, porin regulation, or efflux activity, in shaping resistance phenotypes (58). Furthermore, in *Pseudomonas aeruginosa*, the MexAB-OprM complex, a member of the RND transporter superfamily with broad substrate specificity, can confer antibiotic resistance by actively exporting antimicrobial compounds without the need for specific ARGs (59). In *Klebsiella pneumoniae*, repression of the major outer membrane porin OmpK36 increases resistance to cefazolin and ceftazidime, while also enhancing susceptibility to neutrophil-mediated phagocytosis, potentially influencing pathogenicity (60). Moreover, the outer membrane of Gram-negative bacteria, primarily composed of inner leaflet phospholipids and outer leaflet LPS, serves as a physical barrier against toxic compounds (61). The resistance of the E70 strain may instead arise from its unusual lipid repertoire, specifically the presence of sphingolipids, a rare prokaryotic lipid class with potential roles in antibiotic tolerance (62).

In eukaryotic systems, lipid rafts are functional microdomains enriched in rigid lipids, such as sphingolipids and cholesterol, serving as platforms for membrane-associated signaling and trafficking (63). The presence of lipid rafts, which influence membrane fluidity and stability, suggests that bacterial membranes may also contain lipid raft-like regions known as functional membrane microdomains (FMMs) (64). These membrane domains can locally regulate specific cellular functions, such as protein sorting, signal transduction, and stress responses, in a manner analogous to eukaryotic systems (65, 66). Moreover, FMM formation in bacteria involves the biosynthesis and clustering of isoprenoid-based membrane lipids, which may include bacterially derived sphingolipids as structural components (67). In the human pathogen methicillin-resistant *Staphylococcus aureus* (MRSA), FMMs are formed through the preferential binding and oligomerization of the scaffold protein flotillin within membrane microdomains, and their disassembly has been shown to reduce resistance to β-lactam antibiotics (68). In addition to their role in antibiotic resistance, lipid raft regions are also associated with membrane curvature and stress relief, suggesting a potential link to vesiculation (69). When CAMPs such as PMB and colistin penetrate the lipid domains of bacterial FMMs, they can alter membrane lipid composition and curvature, thereby promoting OMV formation (Fig. 2 and 5). Although lipid A is the primary binding target of colistin, studies in *Caulobacter* have demonstrated that anionic sphingolipids, such as ceramide phosphoglycerate (CPG) and CPG2, can also serve as interaction sites, and defects in these lipids may further influence membrane surface architecture (51). Furthermore, FAME analysis of *P. aeruginosa* OMVs revealed an enrichment of longer and more saturated fatty acids compared to the outer membrane (70). This finding suggests that saturated lipid-enriched outer membrane microdomains, such as those containing sphingolipids, may serve as preferred sites for vesiculation. The preferential enrichment of saturated lipids in OMVs and their association with microdomains implies that sphingolipid-rich regions may function as hotspots for OMV formation, indicating that bacteria, and even eukaryotic cells, possessing sphingolipids may have an increased propensity for vesiculation (Table S1; Fig. 3 and 5D).

While sphingolipid-mediated OMV formation remains poorly characterized, OMV biogenesis may serve not only as a response to antibiotic stress but also as a strategy for host interaction and environmental survival (71). The released OMVs play diverse ecological roles, such as distributing extracellular enzymes that degrade complex substrates, sequestering iron, and acting as decoys for bacteriophages or antibiotics, thereby functioning as public goods within bacterial communities (72, 73). In numerous Gram-negative bacteria, including *E. coli* and *A. baumannii*, OMVs act as decoys for bacteriophages and polymyxins by displaying outer membrane components on their surface: bacteriophages recognize LPS via long-tail fibers, whereas polymyxins interact with lipid A exposed on the vesicle surface (7, 74). Furthermore, under oxidative stress, pathogens, such as *Neisseria meningitidis* and *P. aeruginosa*, increase OMV production (75, 76). In *N. meningitidis*, although the mechanism is not well defined, cultivation at 150% air saturation increased OMV output approximately fourfold. In *P. aeruginosa*, the long O-antigen B-band LPS increases negative surface charge and electrostatic repulsion under oxidative stress, forming B-band–rich domains that drive curvature and membrane scission (75, 76). In nutrient-limited environments, such as the intrahost milieu, *E. coli* O157:H7 increases OMV production and incorporates virulence factors such as Shiga toxin 2a, highlighting the cytotoxic potential of OMVs (77). To represent nutrient limitation faced by many environmental isolates, a relatively low-nutrient R2A medium was chosen, while LB or BHI served as nutrient-rich conditions (78). Upon PMB exposure, expression of the *spt* gene encoding a sphingolipid synthase increased in R2A, but not in LB or BHI (Fig. S6). OMV induction was observed only in R2A, suggesting that sphingolipid-mediated vesiculation may be associated with lipid metabolic pathways modulated by nutritional conditions during PMB treatment, although the underlying mechanisms remain to be elucidated (79, 80).

Although limited to *Pseudomonas putida* species, higher levels of saturated and long-chain fatty acids (e.g., C16:0 and C18:0) have been found in OMVs than in cells, suggesting a more hydrophobic vesicle surface that promotes autoaggregation, biofilm initiation, and adhesion to other cell surfaces (81). The mechanisms by which OMVs interact with or enter host epithelial cells have been partially characterized, with well-established pathways including endocytic routes such as lipid raft– and cholesterol-enriched microdomains, macropinocytosis, clathrin-mediated endocytosis, and caveolin-mediated endocytosis (82). In A549 and T84 epithelial cell lines, OMVs from *Campylobacter jejuni* and *P. aeruginosa* are taken up via lipid raft–dependent pathways, indicating that OMVs can directly engage host cell membranes (83, 84). Because sphingolipids are largely confined to eukaryotic membranes, whether sphingolipid-rich bacterial OMVs can fuse with similarly sphingolipid-enriched eukaryotic membranes has not yet been demonstrated and warrants further investigation (12). Nevertheless, studies in *Saccharomyces cerevisiae* have shown that vacuolar membrane fusion requires specific sphingolipid species, supporting the idea that sphingolipids can act as positive regulators of membrane fusion (85, 86). Bacterially derived sphingolipids within *Porphyromonas gingivalis* membrane vesicles have been shown to suppress cytokine responses in immune cells, thereby modulating host immunity and promoting pathogenesis (87, 88). Given that sphingolipids participate in both host interaction and vesicle formation, their contribution to OMV biogenesis under stress conditions was examined using myriocin, an inhibitor of sphingolipid biosynthesis. Myriocin treatment markedly decreased OMV production even under PMB-induced stress, underscoring the essential role of sphingolipids in vesicle formation (Fig. 5). However, the precise molecular mechanisms linking sphingolipid metabolism to OMV biogenesis and cargo selection remain to be elucidated. Collectively, these findings identify sphingolipids as key mediators of OMV formation and host interaction and suggest that perturbing sphingolipid metabolism could offer a novel strategy to mitigate antibiotic tolerance and host–pathogen communication in MDR bacteria.

**Fig 6:**
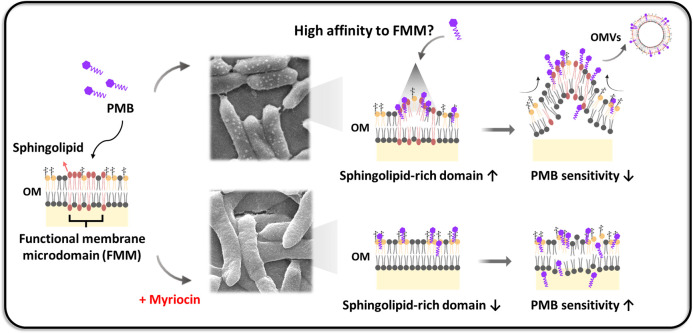
In *Sphingobacterium detergens*, sphingolipid-enriched FMMs create locally ordered, low-permeability regions on the outer leaflet. Our data support a model in which PMB interacts more readily with these sphingolipid-rich patches than with bulk lipid A, likely due to the anionic headgroups and long acyl chains of bacterial sphingolipids. PMB binding at these sites can generate local membrane expansion and curvature, promoting the budding of small OMVs. Disruption of sphingolipid biosynthesis by myriocin weakens these microdomains, resulting in increased PMB susceptibility.

Although it remains uncertain whether PMB interacts with sphingolipid-enriched functional membrane microdomains (FMMs) with higher affinity than with lipid A, our data suggest that such interactions are plausible (Fig. 6). The pronounced increase in PMB susceptibility following myriocin treatment, which inhibits sphingolipid biosynthesis and disrupts FMM integrity, indicates that these microdomains play a critical role in PMB resistance. We propose that sphingolipid-rich FMMs protect the membrane by maintaining local lipid order, reducing permeability, and modulating surface charge distribution. Upon PMB binding, the negatively charged headgroups and long hydrophobic acyl chains of sphingolipids may promote tight association with the outer leaflet, inducing local surface expansion and curvature stress that drive the formation of numerous small, hollow OMVs—analogous to quinolone-induced vesiculation (89). This model highlights the potential role of sphingolipid-containing microdomains as both protective and vesiculogenic platforms during cationic peptide stress, a hypothesis that warrants further mechanistic investigation.

## MATERIALS AND METHODS

### Bacterial strains and growth conditions

*Sphingobacterium detergens* E70 was initially grown in Reasoner’s 2A (R2A) medium at 30°C (17). For all experiments, overnight cultures were diluted 1:100 into fresh R2A broth and incubated for 4 h at 30°C with shaking (180 rpm), followed by treatment with PMB at the indicated concentration for an additional 3 h under the same conditions. Where applicable, PMB was used at one-quarter of the MIC (1/4 MIC, 64 µg/mL), as determined by standard broth microdilution (2). The sub-inhibitory concentration corresponding to one-quarter of the MIC was selected as it produced distinct membrane alterations while maintaining cell viability. Our SEM analysis showed that vesiculation gradually decreased with lower PMB concentrations (Fig. S13). For experiments involving myriocin, the inhibitor was added at the time of 1:100 dilution so that cells were exposed from the beginning of the growth, thereby suppressing sphingolipid biosynthesis throughout the assay.

### Measurement of MIC

Antibiotic susceptibility tests were conducted using the broth dilution method in 96-well plates to determine the susceptibility of the E70 strain (2). All antibiotics were purchased from Sigma-Aldrich (USA), and the following concentrations were assessed: PMB (1–512 µg/mL), colistin (1–512 µg/mL), azithromycin (1–512 µg/mL), ampicillin (1–512 µg/mL), meropenem (1–512 µg/mL), gentamicin (1–512 µg/mL), tetracycline (1–512 µg/mL), doxycycline (1–512 µg/mL), and oxytetracycline (1–512 µg/mL). All isolates were cultured O/N in R2A broth, then diluted (1:100) into fresh R2A broth (5 mL), and incubated until they reached the early exponential phase (OD_600_ = 0.4). The MIC values were determined by optical density (OD₆₀₀) measurement and visual inspection after 24 h of incubation. An OD₆₀₀ value below 0.05, corresponding to no visible growth, was used as the cutoff for MIC determination. The cultured cells were subsequently transferred into a 96-well plate containing each antibiotic, and the final cell numbers were adjusted to approximately 10^6^ CFU/ml. Incubation was continued at 37°C for 24 h to calculate the MIC.

### Phylogenetic analysis

A total of 62 *Sphingobacterium* reference genomes were retrieved from the NCBI RefSeq database. To assess the genome-level relatedness between these strains and *S. detergens* E70, a whole-genome distance-based phylogenetic tree was constructed using Mashtree v1.4.6. ARG profiling was performed on the same set of genomes using the ResFinder database (https://cge.food.dtu.dk/services/ResFinder/), implemented in CLC Genomics Workbench version 22 (Qiagen). The detection thresholds were set to a minimum nucleotide identity of 30% and a minimum alignment length of 50% relative to the reference gene, following the criteria described previously (17). In addition, basic genomic features, such as genome size and GC content, were calculated using the DFAST pipeline (v1.2.18). IS elements were also annotated to assess potential contributions of MGEs to genomic plasticity. Comparative analyses across genomes were subsequently integrated with ARG distribution and IS element profiles to interpret the evolutionary context of resistance in the genus *Sphingobacterium*.

### Measurement of cell surface components

For biofilm formation, overnight cultures of *S. detergens* E70 were diluted 1:100 into fresh R2A broth and incubated at 30°C with shaking for 4 h to reach the early exponential phase. Cells were then treated with PMB (1/4 MIC, 64 µg/mL) for 3 h under the same conditions. Subsequently, 200 µL aliquots of each culture were transferred into sterile, flat-bottom 96-well polystyrene plates (Corning, USA) and incubated under static conditions at 30°C for 24 h to allow biofilm formation. After incubation, planktonic cells were gently removed, and wells were washed three times with sterile distilled water to remove loosely attached cells (90). Biofilms were stained with 200 µL of 0.1% (wt/vol) crystal violet solution for 20 min at room temperature. Excess dye was removed by rinsing the wells three times with distilled water, and plates were air-dried completely. Bound dye was solubilized with 200 µL of 95% ethanol for 15 min, and absorbance was measured at 570 nm using a microplate reader (TECAN, Switzerland). Each condition was tested in triplicate, and the results are presented as the mean ± standard deviation.

For three-dimensional biofilm visualization, *S. detergens* E70 biofilms were grown in confocal dishes under the same conditions as described for biofilm quantification (SPL Life Sciences, Republic of Korea). Following incubation, wells were gently washed twice with sterile phosphate-buffered saline (PBS) to remove non-adherent cells. Biofilms were stained with 200 µL of FilmTracer SYPRO Ruby (Invitrogen, USA) according to the manufacturer’s instructions and incubated 30 min at room temperature in the dark, labeling proteinaceous matrix components through non-covalent interactions with basic residues (lysine, arginine, and histidine) and with the polypeptide backbone. Excess dye was carefully removed, and the wells were washed three times with sterile PBS using a peristaltic pump at a flow rate of 2 µL/min to minimize disruption of the fluorescently labeled biofilm structure. Stained biofilms were imaged using a confocal laser scanning microscope (LSM 700, Carl Zeiss, Germany) equipped with a 100× oil-immersion objective. Excitation was performed at 450–500 nm, and emission was collected at 610–650 nm. Z-stacks were acquired at 1 µm intervals through the entire biofilm depth, and three-dimensional reconstructions were generated using Zen 2.1 Blue Edition software (Carl Zeiss). All samples were imaged under identical acquisition settings.

Measurements were carried out using a Zetasizer Nano ZS (Malvern Instruments) with standard settings at 25°C. To detect membrane-bound vesicles, FM4-64 (Thermo Fisher) was added to cultures at a final concentration of 2 µg/mL and incubated for 15  min in the dark. Cells were washed twice with PBS, mounted on glass slides, and imaged using a CLSM (LSM 800, Carl Zeiss, Germany) under identical acquisition settings for all samples.

### Electron microscopy analysis

For electron microscopy analyses, *S. detergens* E70 cells were cultured overnight in R2A broth at 30°C, diluted 1:100 into fresh medium (10 mL), and incubated until mid-exponential phase. For antibiotic treatment, cells were exposed to one-quarter of the MIC (1/4 MIC) for 3 h and subsequently harvested by centrifugation at 13,000 rpm for 10 min. The cell pellets were washed twice in PBS (pH 7.2) and fixed in 2.5% glutaraldehyde prepared in 0.1 M phosphate buffer (pH 7.2) at 4°C for 4 h. Following primary fixation, cells were centrifuged again at 13,000 rpm for 1 min, the supernatant was removed, and the pellets were washed three times with PBS. Secondary fixation was performed using a 1% osmium tetroxide solution at 4°C for 2 h, which also acts as a post-fixation stain to enhance membrane contrast for electron microscopy, followed by repeated washing with PBS. The fixed samples were dehydrated in a graded ethanol series (30%, 50%, 70%, 80%, 90%, and three times with 100%) for 10 min at each step, with centrifugation between steps to remove the supernatant. The ethanol-dehydrated samples were resuspended in 30 µL of 100% ethanol, and 5 µL aliquots were spotted onto aluminum stubs, air-dried, and sputter-coated with platinum. The specimens were observed under a field-emission scanning electron microscope (Quanta 250; FEI, USA).

For thin-section TEM, cells were prepared as described above for primary fixation. Fixed cells were embedded in resin, sectioned with an ultramicrotome, mounted on copper grids coated with carbon film, and post-stained with 2% uranyl acetate. For negative-stain TEM, aliquots of fixed cells were adsorbed onto carbon-coated copper grids, washed briefly with distilled water, and stained with 2% uranyl acetate. TEM observations were conducted using a JEM-1011 system (JEOL, USA).

### Bacterial cell observation using microscopy

Dansyl-PMB, previously described in a prior study, was used to visualize PMB binding to the cell surface (7). Briefly, PMB sulfate (40 mg; Sigma-Aldrich, USA) was dissolved in 1.2 mL of 0.1 M NaHCO₃ buffer, and dansyl chloride (10 mg; Sigma-Aldrich, USA) was dissolved in 0.8 mL of acetone. The dansyl chloride solution was added dropwise to the PMB solution and gently agitated in the dark at room temperature for 90 min to ensure complete mixing during the reaction. The reaction mixture was then applied to a Sephadex G-50 column (50 × 2.5 cm; Sigma-Aldrich, USA) pre-equilibrated with 10 mM sodium phosphate buffer (pH 7.1) containing 0.145 M NaCl. Fractions of 5–6 mL were collected and examined under a handheld UV lamp to distinguish dansyl-PMB (yellow fluorescence) from unreacted dansyl chloride (blue-green fluorescence). Fractions containing dansyl-PMB were extracted with an equal volume of n-butanol, and the organic phase was evaporated to dryness in a glass Petri dish inside a desiccator at 37°C for 24 h. The dried dansyl-PMB was reconstituted in 3 mL of 5 mM HEPES buffer (pH 7.0) and stored at −20°C until use.

For bacterial staining, *S. detergens* E70 and *A. baumannii* ATCC 17978 were cultured in 5 mL R2A medium to the early exponential phase. Cells were harvested by centrifugation at 13,000 rpm for 10 min, washed twice with PBS (pH 7.2), and resuspended in 1 mL of PBS. Dansyl-PMB was added to a final concentration of 2 µg/mL, and the suspensions were incubated at 37°C for 30 min in the dark to prevent photobleaching. After incubation, the cells were washed twice with PBS to remove unbound dye and mounted on glass slides for imaging. CLSM was performed using an LSM 700 microscope (Carl Zeiss, Germany). Fluorescence images were acquired with excitation at 330 nm and emission at 540 nm, and the resulting images were analyzed and processed using Zen 2.1 software (Blue edition; Carl Zeiss, Germany). FM4-64 dye was added to a final concentration of 2 µg/mL, and cells were incubated for 15 min at room temperature in the dark to prevent photobleaching. After staining, cells were washed twice with PBS, mounted on glass slides, and immediately imaged by CLSM using an LSM 700 microscope (Carl Zeiss, Germany). Fluorescence was recorded using excitation/emission wavelengths of 510/610 nm under identical acquisition settings for all samples. For OMV quantification, cell-free supernatants were obtained by centrifugation (12,000 × *g*, 1 min) and filtration through 0.22 µm syringe filters (Merck Millipore, USA). A 200 µL aliquot of the supernatant was transferred to a black 96-well plate (SPL Life Sciences, Korea), mixed with FM4-64 at 2 µg/mL, and fluorescence was measured using a TECAN plate reader (Switzerland) at 510/610 nm with 5 nm slit widths. These fluorescence intensities were used as a relative measure of OMV abundance.

### Bacterial membrane fractionation

Membrane fractionation was performed to isolate the inner membrane, outer membrane, and periplasmic contents of *S. detergens* E70. Cells were cultured in R2A medium and harvested by centrifugation at 6,000  ×  *g* for 10 min at 4°C and washed twice with ice-cold PBS. For periplasmic extraction, cell pellets were resuspended in 20 mM Tris-HCl (pH 8.0) containing 20% sucrose and 1 mM EDTA, followed by incubation on ice for 10 min. After centrifugation, the pellet was resuspended in cold water and incubated for 10 min to induce osmotic shock. The resulting supernatant was collected as the periplasmic fraction. Isolation of inner and outer membrane proteins was performed based on a previously described method (4). Briefly, harvested cell pellets were suspended in a hypertonic solution consisting of 0.5 M sucrose and 10 mM Tris-HCl (pH 7.5) supplemented with EDTA. The suspension was chilled on ice and treated with lysozyme to weaken the cell wall. Specifically, 180 µL of lysozyme (10 mg/mL) was mixed into the suspension and incubated for 2 min, followed by the addition of 12.5 mL of 1.5 mM EDTA and a further 7-min incubation on ice with gentle mixing. Cells were then collected by centrifugation at 9,000 × *g* for 10 min at 4°C, and the resulting pellets were retained. These pellets were resuspended in 25 mL of a secondary buffer containing 0.2 M sucrose and 10 mM Tris-HCl (pH 7.5), to which 55 µL of 1 M MgCl₂, 1 µL of RNase/DNase enzyme mix, and 1 µL of protease inhibitor cocktail were added. The suspension was disrupted by probe sonication on ice, and cell lysates were clarified by centrifugation at 7,000 × *g* for 10 min at 4°C. The pellet containing membrane material was finally resuspended in a 20% (wt/vol) sucrose solution prepared with 1 mM EDTA and 10 mM Tris-HCl (pH 7.5) for subsequent density-gradient separation. A discontinuous sucrose gradient composed of three layers (3 mL of 73%, 5 mL of 45%, and 1 mL of 20% [wt/vol] sucrose) was prepared, and the membrane suspension was carefully layered on top of the 20% sucrose fraction, followed by ultracentrifugation at 140,000 × *g* for 15 h at 4°C using a Beckman SW 40 Ti rotor. The inner and outer membrane protein fractions were collected from the upper and lower layers, respectively, washed with PBS, and pelleted again at 300,000 ×  *g* for 90  min at 4°C before resuspension. For membrane fractionation experiments, proteins from inner, outer, and periplasmic fractions were prepared as described above and resolved on SDS-PAGE gels (12%). In this case, gels were stained with Coomassie Brilliant Blue R-250 for 1 h and destained with 40% methanol/10% acetic acid until a clear background was obtained. Protein ladders (PageRuler Prestained Protein Ladder, Thermo Fisher Scientific, USA) were included in all runs. Banding patterns were documented using an iBright imaging system (Thermo Fisher Scientific, USA).

### OMV purification

OMVs were quantified using the lipophilic dye FM 4-64 (Invitrogen, USA) as previously described (7, 91). Briefly, *S. detergens* E70 was cultured overnight in R2A medium at 30°C, diluted 1:100 into fresh medium, and incubated for 6 h at 30°C with shaking (190 rpm) to reach the exponential phase. Cultures were centrifuged at 12,000 ×  *g* for 1  min, and the resulting supernatants were filtered through a 0.22 µm syringe filter (Merck Millipore) to remove residual cells and debris. A 200 µL aliquot of the filtered supernatant was transferred to a black 96-well microplate (SPL Life Sciences), and FM4-64 was added to a final concentration of 2 µg/mL. Fluorescence was measured using a plate reader (TECAN, Switzerland) at excitation/emission wavelengths of 510/610 nm, with slit widths set to 5  nm. For OMV purification, 2 L of the E70 cell culture was prepared under the same conditions. After centrifugation and filtration, the supernatant was concentrated using a Vivaspin 20 centrifugal concentrator (100 kDa MWCO) at 5,000 ×  *g* and 4°C. The OMV-enriched concentrate was subjected to ultracentrifugation at 300,000 ×  *g* for 90 min at 4°C using a Beckman Optima XE-90 ultracentrifuge. The resulting pellet was resuspended in 50% (wt/vol) iodixanol and layered onto a discontinuous Optiprep (iodixanol) gradient (10%–45% [wt/vol]). The OMV-containing fraction was pelleted again by ultracentrifugation (300,000 ×  *g*, 90 min, 4°C) and resuspended in 1 mL PBS. Purified OMVs were quantified by FM4-64 staining and total protein concentration using the Bradford assay. Particle size distribution and concentration were measured using nanoparticle tracking analysis (NTA; NanoSight NS300, Malvern Instruments) with purified OMV samples. Proteins from purified OMVs were analyzed by SDS-PAGE under denaturing conditions. OMV samples were normalized to 50–100 µg total protein (Bradford assay) and mixed with 4× Laemmli sample buffer containing 5% β-mercaptoethanol. Samples were heated at 95°C for 5 min before electrophoresis. Proteins were resolved on a 12% polyacrylamide separating gel with a 5% stacking gel. Electrophoresis was performed at a constant voltage of 80 V through the stacking gel and 120 V through the separating gel until the dye front reached the bottom (~90 min). Following electrophoresis, gels were fixed in 50% methanol and 10% acetic acid for 30 min, washed twice with 50% ethanol, and treated with sensitizing solution (0.02% sodium thiosulfate) for 1 min. After rinsing with distilled water, gels were incubated in 0.1% silver nitrate for 20 min at 4°C, briefly rinsed, and developed in 0.04% formaldehyde in 2% sodium carbonate until the protein bands became visible. The staining reaction was stopped by adding 5% acetic acid. Protein molecular weight standards (PageRuler Prestained Protein Ladder, Thermo Fisher Scientific, USA) were run in parallel. Banding patterns were documented using an iBright imaging system (Thermo Fisher Scientific, USA).

### Flow cytometric analysis of OMVs

To detect OMV-like particles in culture supernatants, *S. detergens* E70 was grown under control or PMB-treated conditions as described above. After incubation, cell-free supernatants were obtained by centrifugation at 12,000 × *g* for 10 min, followed by filtration through 0.22 µm syringe filters (Merck Millipore, USA) to remove residual cells and debris. Aliquots (200 µL) of the filtered supernatants were incubated with FM4-64 (Thermo Fisher Scientific, USA) at a final concentration of 2 µg/mL for 15 min at room temperature in the dark. Fluorescence signals corresponding to vesicle-sized particles were measured using a BD Accuri C6 Plus flow cytometer (BD Biosciences, USA) equipped with a 488 nm laser and a 610/20 nm emission filter. Events were collected at a low flow rate until 10,000 particles were recorded per sample. Data were analyzed using BD Accuri C6 software (v1.0.264.21). Three independent experiments were performed for each condition, and approximately 50,000 particles were analyzed per experiment.

### Quantitative reverse transcription-PCR

Total RNA from the E70 strain was extracted at different growth time points in R2A medium (5 mL) using the RNeasy Mini Kit (Qiagen, Germany). Cells of the E70 strain grown in R2A broth (5 mL per sample) were harvested at the indicated time points by centrifugation (13,000 × *g* for 5 min at 4°C). Cell pellets were resuspended in 100 µL TE buffer (10 mM Tris-HCl, 1 mM EDTA, pH 8.0) containing lysozyme (1 mg/mL) and incubated for 5 min at room temperature to weaken the cell envelope. Buffer RLT supplemented with β-mercaptoethanol (10 µL per 1 mL RLT) was added (350 µL), the suspension was mixed vigorously and homogenized. An equal volume of 70% ethanol (350 µL) was added, mixed, and the lysate was loaded onto an RNeasy spin column. After centrifugation (10,000 × *g* for 15 s), the column was washed with 700 µL Buffer RW1. On-column DNase digestion (RNase-Free DNase Set, Qiagen) was performed by applying 80 µL DNase I mix (10 µL DNase I + 70 µL Buffer RDD) directly onto the membrane and incubating for 15 min at room temperature. The column was washed with 700 µL Buffer RW1, followed by two washes with 500 µL Buffer RPE (first wash spin, 15 s; second wash spin, 2 min). A dry spin (1 min) removed residual ethanol. RNA was eluted in 30–50 µL RNase-free water after a 1- to 2-min incubation on the membrane. Samples were stored at −80°C until use. For quantitative real-time PCR (qRT-PCR), 1,000 ng of total RNA was first treated with DNase I (1 µL; Thermo Fisher Scientific, USA) and then used for cDNA synthesis with the RevertAid Reverse Transcription Kit (Thermo Fisher Scientific, USA). Amplification was performed on a QuantStudio 5 Real-Time PCR System (Applied Biosystems, USA) using Power SYBR Green PCR Master Mix (Applied Biosystems, USA). Each 20 µL reaction mixture contained 10 µL of master mix, 1 µL of forward primer (10 pmol), 1 µL of reverse primer (10 pmol), 2 µL of diluted cDNA (1:10), and 7 µL of nuclease-free water. Cycling conditions were as follows: initial denaturation at 95°C for 10 min, followed by 40 cycles of denaturation at 95°C for 15 s and annealing/extension at 60°C for 1 min. A melt-curve analysis was then performed with steps of 95°C for 15 s, 60°C for 1 min, and 95°C for 15 s. Relative gene expression was normalized to 16S rRNA, which served as the internal reference. Primer sequences are provided in Table S1.

### Fatty acid extraction

FAMEs of *S. detergens* E70 were prepared from mid-exponential phase cells following the standard protocol (92, 93). Briefly, bacterial cell pellets were harvested from 50 mL cultures by centrifugation (14,000 × *g*, 10 min, 4°C) and subjected to saponification in 15% NaOH in 50% methanol at 100°C for 30 min. The hydrolyzed fatty acids were subsequently methylated in 6 N HCl in 50% methanol at 80°C for 10 min to yield FAME derivatives. Following extraction with hexane/methyl tert-butyl ether (1:1, vol/vol), the organic phase was washed with 0.3 N NaOH, dried under nitrogen, and reconstituted in hexane. Samples were analyzed using the MIDI/Hewlett-Packard Microbial Identification System (Agilent, USA) equipped with a gas chromatograph fitted with a fused-silica capillary column. Fatty acid peaks were identified and quantified by comparison with the MIDI Sherlock Microbial Identification System library. Relative abundances were expressed as a percentage of the total fatty acids detected.

### Chromatographic analyses

The E70 strain was cultured in 500 mL of R2A medium at 30°C with shaking until the mid-exponential phase. Cells were collected by centrifugation, and total lipids were extracted at room temperature by shaking in a chloroform:methanol:water mixture (1:2:0.8, vol/vol/vol) for 18 h (47). Following extraction, a 2:1 ratio of chloroform and water was added to the suspension to achieve a final solvent ratio of 1:1:0.9 (chloroform:methanol:water, vol/vol/vol). The mixture was then centrifuged at 1,000 × *g* at room temperature to allow phase separation into three distinct layers. The organic extract was dried, stored at –20°C, and re-suspended in chloroform:methanol (1:2, vol/vol). For LC-MS analysis, lipid extracts were analyzed using an API3200 HPLC system (AB SCIEX, USA). Mass system was Turbo V Source (TurbolonSpray [ESI] + APCI probe). Separation was performed on a BEH C_18_ column (2.1 × 50 mm, 1.7 µm particle size) at a flow rate of 0.4 mL/min. Data acquisition and quantitative analysis were performed using Analyst^®^ 1.4.2 software. The mobile phases consisted of 0.1% formic acid in acetonitrile/water (20:80, vol/vol) (solvent A) and 0.1% formic acid in acetonitrile/2-propanol (20:80, vol/vol) (solvent B). A stepwise gradient was applied as follows: 0.0–1.0 min, 30% B; 1.0–2.5 min, 30–70% B; 2.5–4.0 min, 70–80% B; 4.0–5.0 min, 80% B; 5.0–6.5 min, 80–90% B; and 6.6–7.5 min, 100% B. The column was re-equilibrated at 30% B for 1.4 min. The total run time was 9 min, with an injection volume of 3 µL. For TLC, lipid extracts were spotted onto TLC Silica Gel 60 plates and developed using a solvent system of chloroform:methanol:acetic acid:water (100:20:12:5, vol/vol/vol/vol). Bacterial sphingolipids were identified based on matching Rf values, and lipid spots were visualized using the ninhydrin reagent with subsequent heating (47).

## SUPPLEMENTAL MATERIAL

Supplemental material aem.01726-25-s0001.pdf Figures S1 to S13; Tables S1 and S2.

ASM does not own the copyrights to Supplemental Material that may be linked to, or accessed through, an article. The authors have granted ASM a non-exclusive, world-wide license to publish the Supplemental Material files. Please contact the corresponding author directly for reuse.
