# Supplementary material for: Sphingolipid-mediated vesiculation in multidrug-resistant Sphingobacterium detergens under polymyxin B stress
Source: Appl Environ Microbiol. 2025 Dec 3;91(12):e01726-25. doi: 10.1128/aem.01726-25 (PMC12724326; doi:10.1128/aem.01726-25)
Supplement: Supplemental material — Figures S1 to S13; Tables S1 and S2. [file aem.01726-25-s0001.pdf]

Sphingolipid-mediated vesiculation in multidrug-resistant *Sphingobacterium detergens* under polymyxin B stress

Jihyeon Min[a], Yewon Woo[a], Yerim Park[a], and Woojun Park*

Laboratory of Molecular Environmental Microbiology, Department of Environmental Science and Ecological Engineering, Korea University, Seoul, 02841, Republic of Korea.

[a] These authors contributed equally to this work.

Keywords: Multi-drug resistance; polymyxin; lipid raft; microdomain; outer membrane vesicle; cationic peptide.

***Corresponding author:** Dr. Woojun Park, Department of Environmental Science and Ecological Engineering, Korea University, Seoul 02841, Republic of Korea.

E-mail: wpark@korea.ac.kr

Fax: +82-2-953-0737

Phone: +82-2-3290-3067

Figure S1. Antibiotic susceptibility profiles of *Sphingobacterium detergens* E70 with or without myriocin**. (A)** Heatmap representation of minimum inhibitory concentrations (16–512 µg/mL) for nine antibiotics without myriocin tested against the animal feces-derived *S. detergens* E70 strain. **(B)** Heatmap representation of minimum inhibitory concentrations (2–512 µg/mL) for nine antibiotics treated with myriocin tested against the animal feces-derived *S. detergens* E70 strain. Antibiotics included polymyxin B (PMB), colistin (COL), azithromycin (AZM), ampicillin (AMP), meropenem (MPN), gentamicin (GEM), tetracycline (TET), doxycycline (DXC), and oxytetracycline (OTC). Each cell corresponds to the growth response at the indicated antibiotic concentration, with darker red shading representing stronger inhibition. Asterisks denote the MIC values determined for each antibiotic.

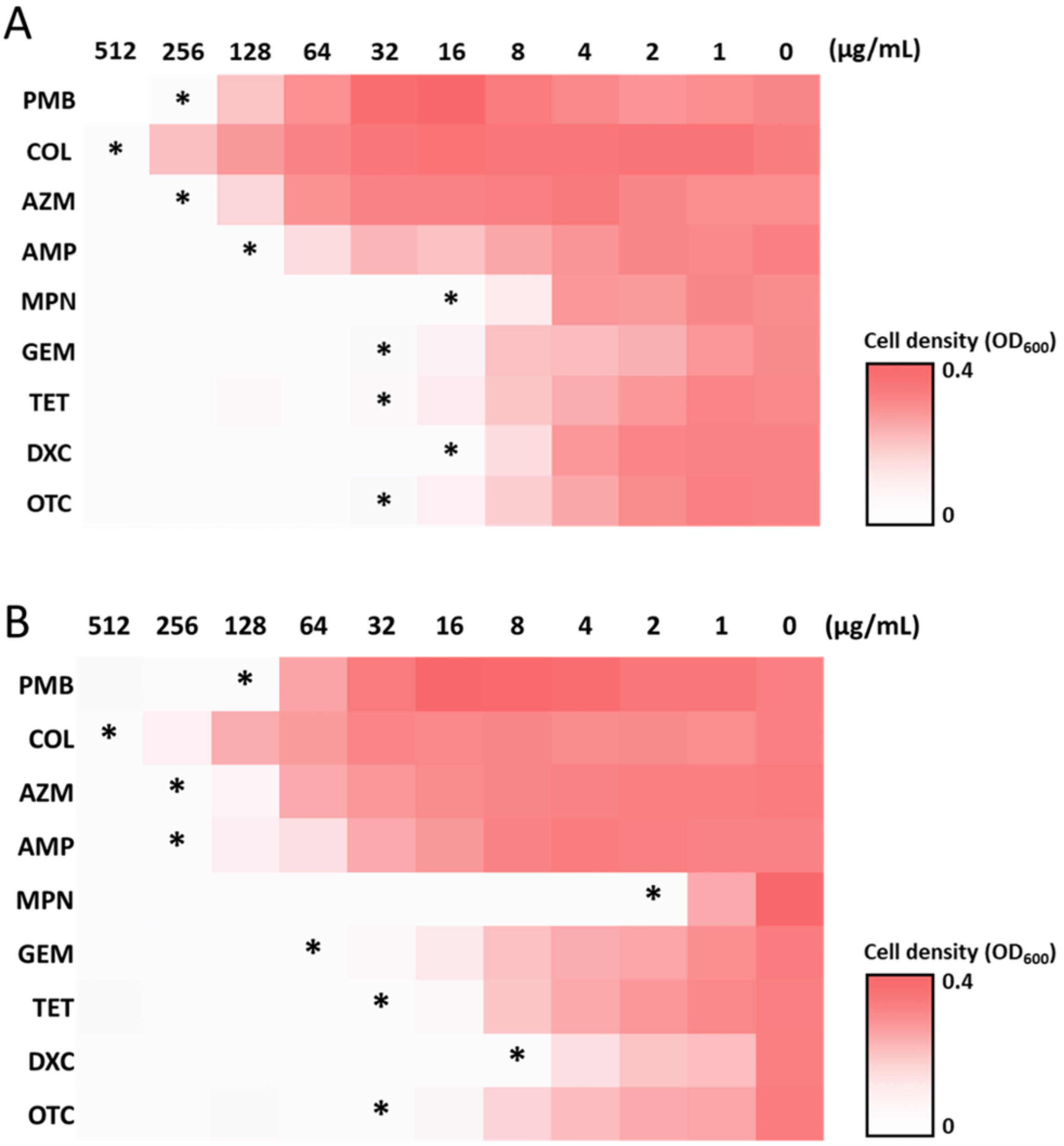

Figure S2. Phylogenetic analysis of *Sphingobacterium* and *Acinetobacter* genomes. Whole-genome phylogeny was constructed using the Mashtree tool based on available reference genomes. The left panel shows 62 *Sphingobacterium* genomes, with the feces-derived *S. detergens* E70 strain highlighted in yellow. The right panel displays 154 *Acinetobacter* genomes, including *A. baumannii* (red), *A. calcoaceticus* and *A. oleivorans* (blue), with other members shown in black. Branch lengths represent genetic distances, and the tree scale indicates the number of nucleotide substitutions per site.

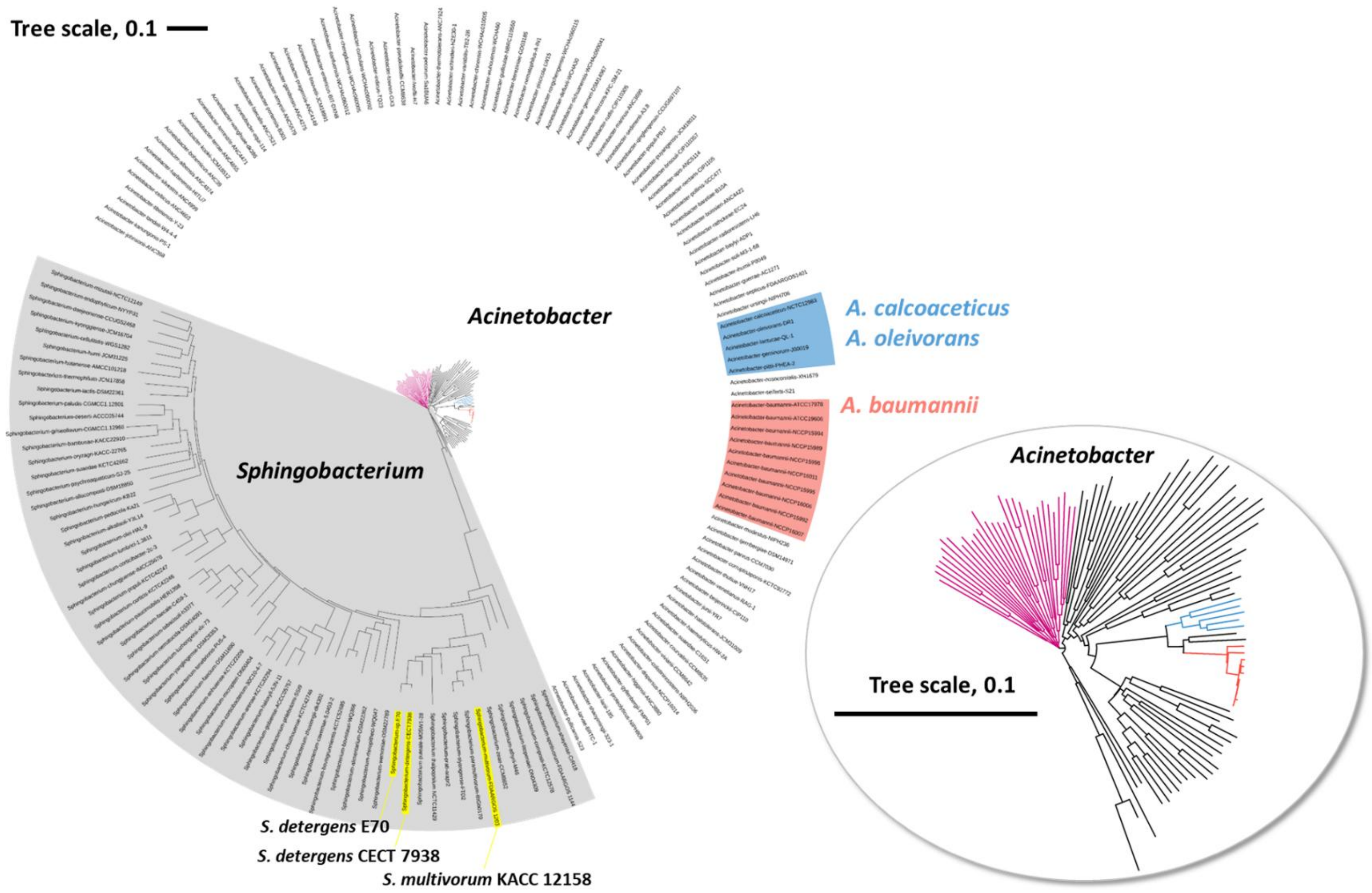

Figure S3. Comparative abundance of antibiotic resistance genes (ARGs) and insertion sequence (IS) elements across bacterial taxa. Distribution of ARGs (red bars, left) and IS families (blue bars, right) across representative Gram-negative pathogens and environmental bacteria. Each bar represents the average number of genes or elements identified per genome within each genus or species.

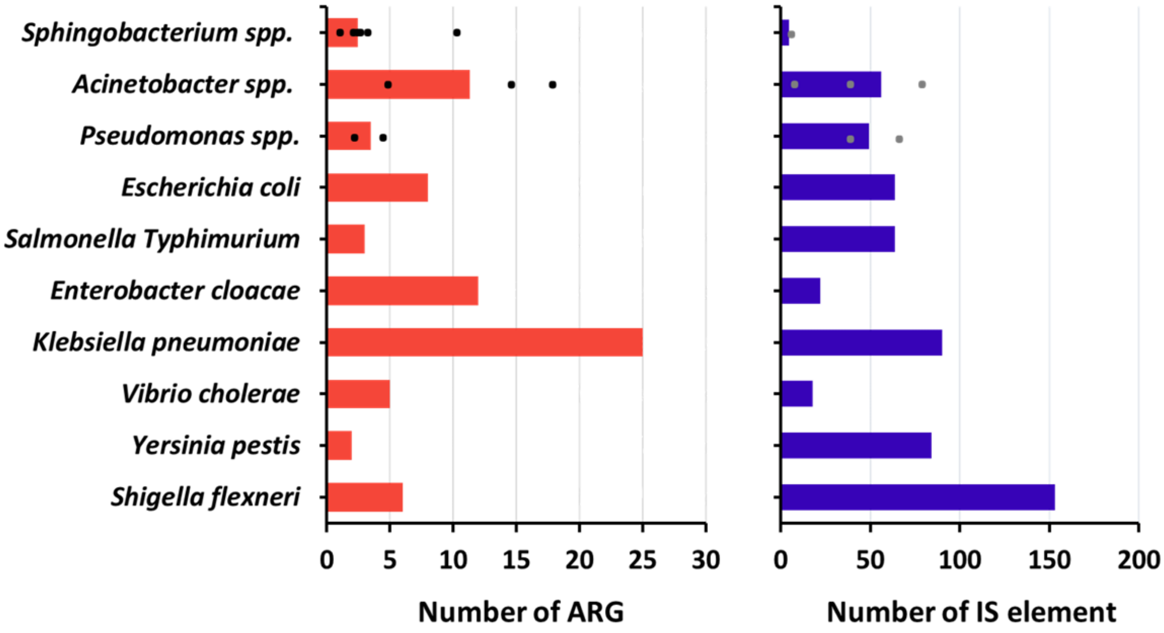

Figure S4. Scanning electron microscopy analysis of *S.detergens* E70 under antibiotic treatment. Representative SEM images of E70 cells exposed for 3 h during the early exponential phase to different antibiotics at ¼ MIC.

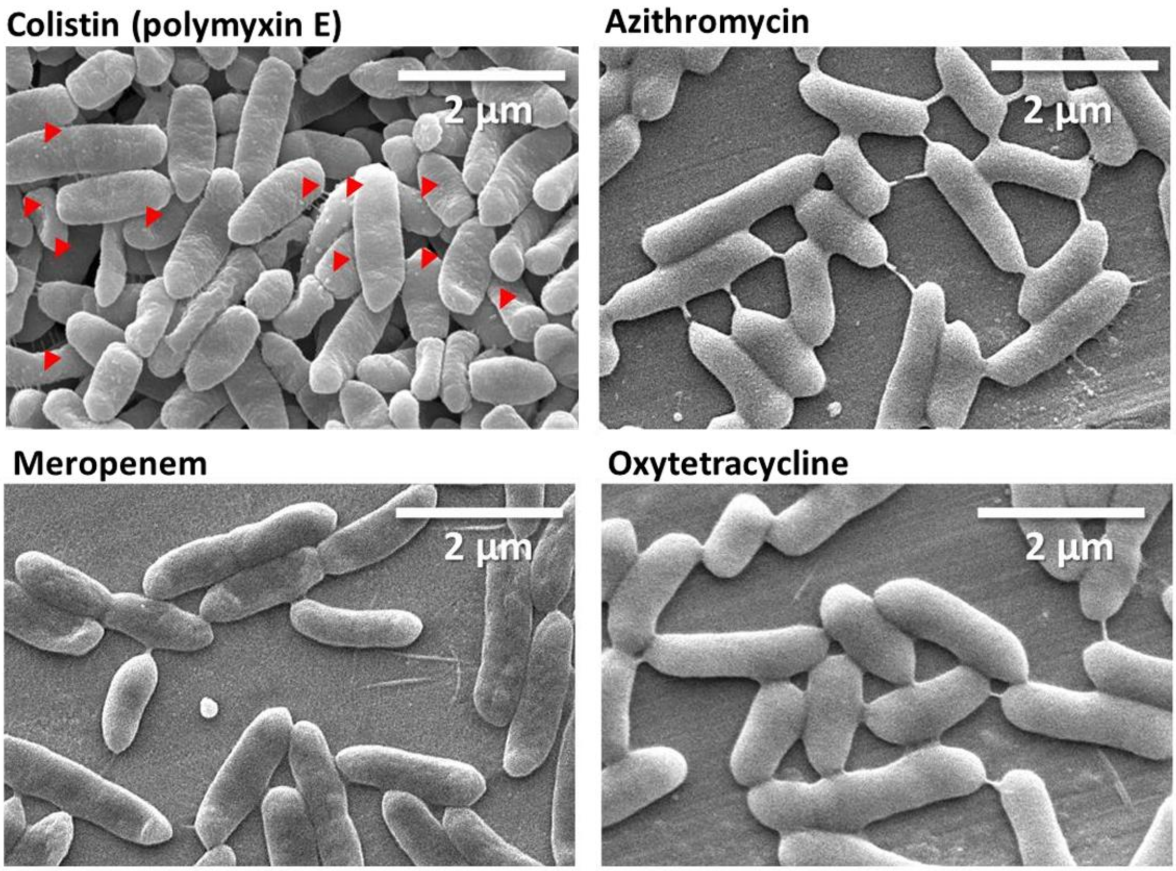

Figure S5. Growth and membrane morphology of *S. detergens* E70 in different nutrient media. **(A)** Growth curves of strain E70 in R2A, LB, and BHI media. Doubling times (μ) were calculated from 6–12 h growth phases. Growth in R2A medium was used as the control condition. **(B)** Representative SEM images of E70 cells cultured in BHI and LB media with PMB treatment.

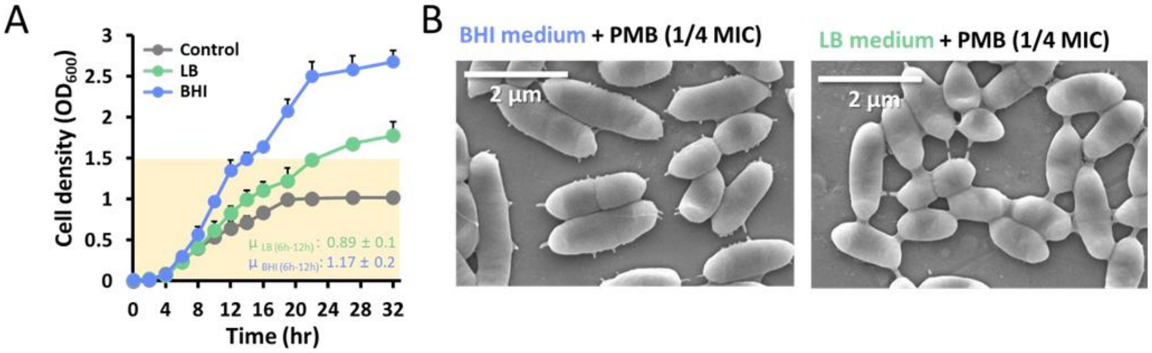

Figure S6. Sphingolipid biosynthesis pathway and transcriptional responses of the *spt* and *eptA* genes under PMB treatment. **(A)** Proposed bacterial sphingolipid biosynthetic pathway in *Sphingobacterium detergens*. **(B)** Quantitative RT-PCR analyses of *spt* gene expression in the E70 strain following exposure to polymyxin B (PMB, ¼ MIC) for 0, 60, 90, and 120 min, showing progressive upregulation relative to untreated controls. **(C, D)** Comparative *spt* expression across media (R2A, LB, BHI) after PMB treatment**. (E)** Comparative *eptA* expression under the control and PMB treatment conditions (*, $p < 0.5$; **, $p < 0.01$; ***, $p < 0.005$; ****, $p < 0.001$; *NS*, no significant).

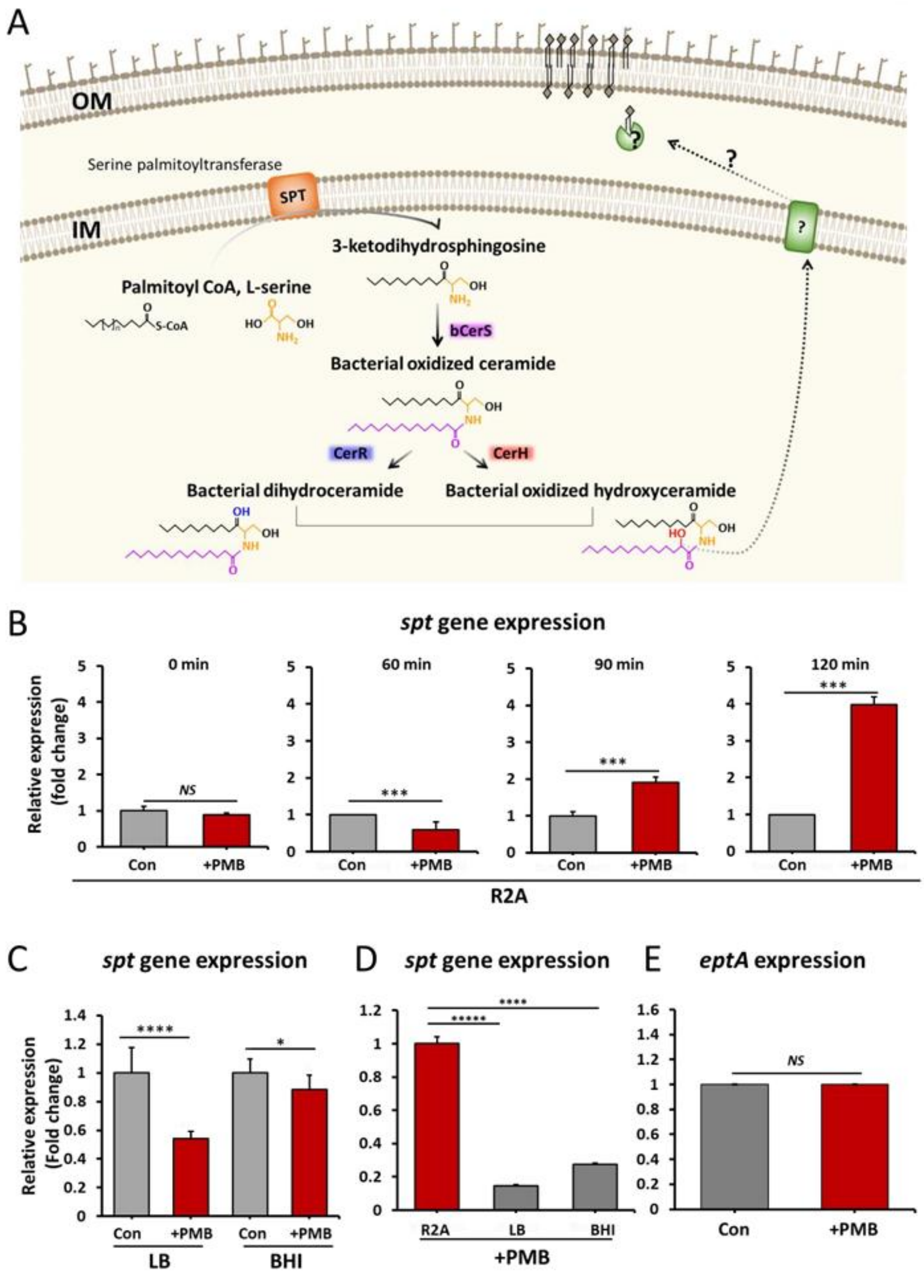

Figure S7. Protein-poor composition of OMVs from the E70 strain. **(A)** Representative images of purified OMV fraction obtained from control and PMB-treated cultures. **(B)** SDS-PAGE analysis of OMVs purified under control and PMB-treated conditions. No distinct protein bands were detected, even at a loading concentration of 100 μg/mL, and only faint smear-like patterns were visible.

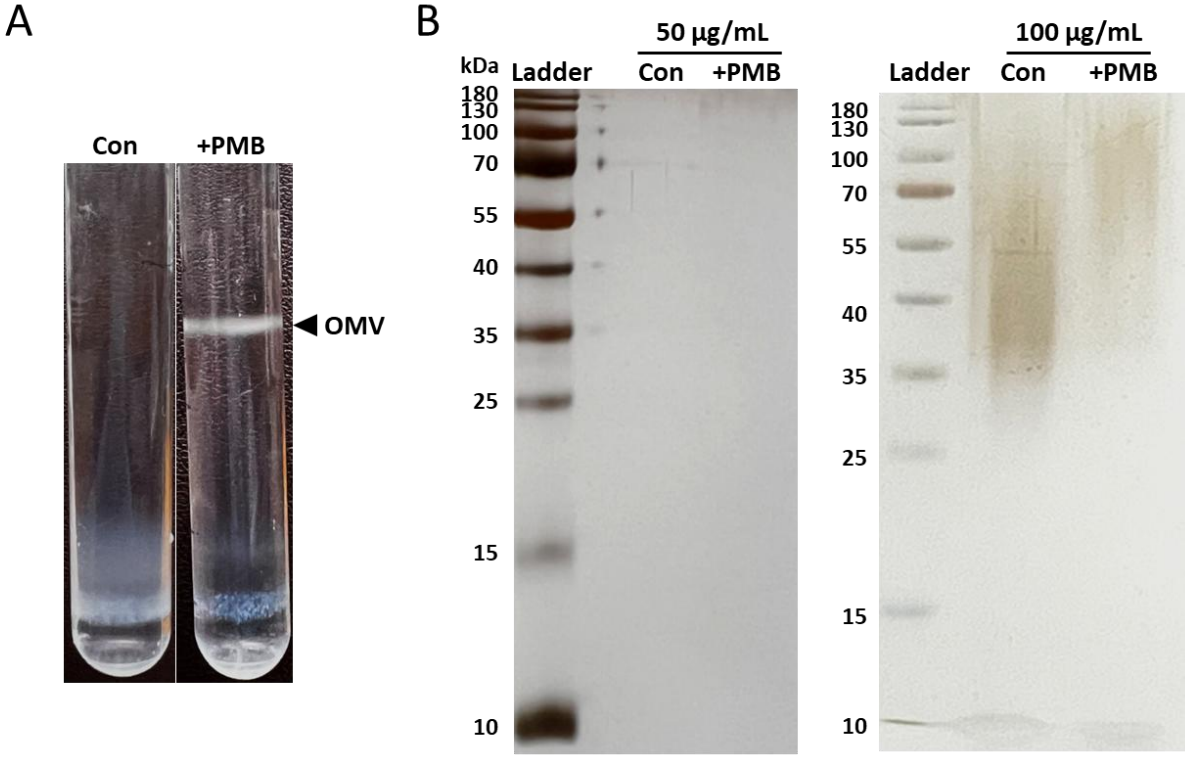

Figure S8. Time-dependent surface alterations of *S. detergens* E70 under PMB treatment. Cells were exposed to polymyxin B (PMB) at ¼ MIC in R2A medium and examined by scanning electron microscopy (SEM) at different time points (15, 30, 60, and 120 min; red arrowheads indicate clearly visible vesicles).

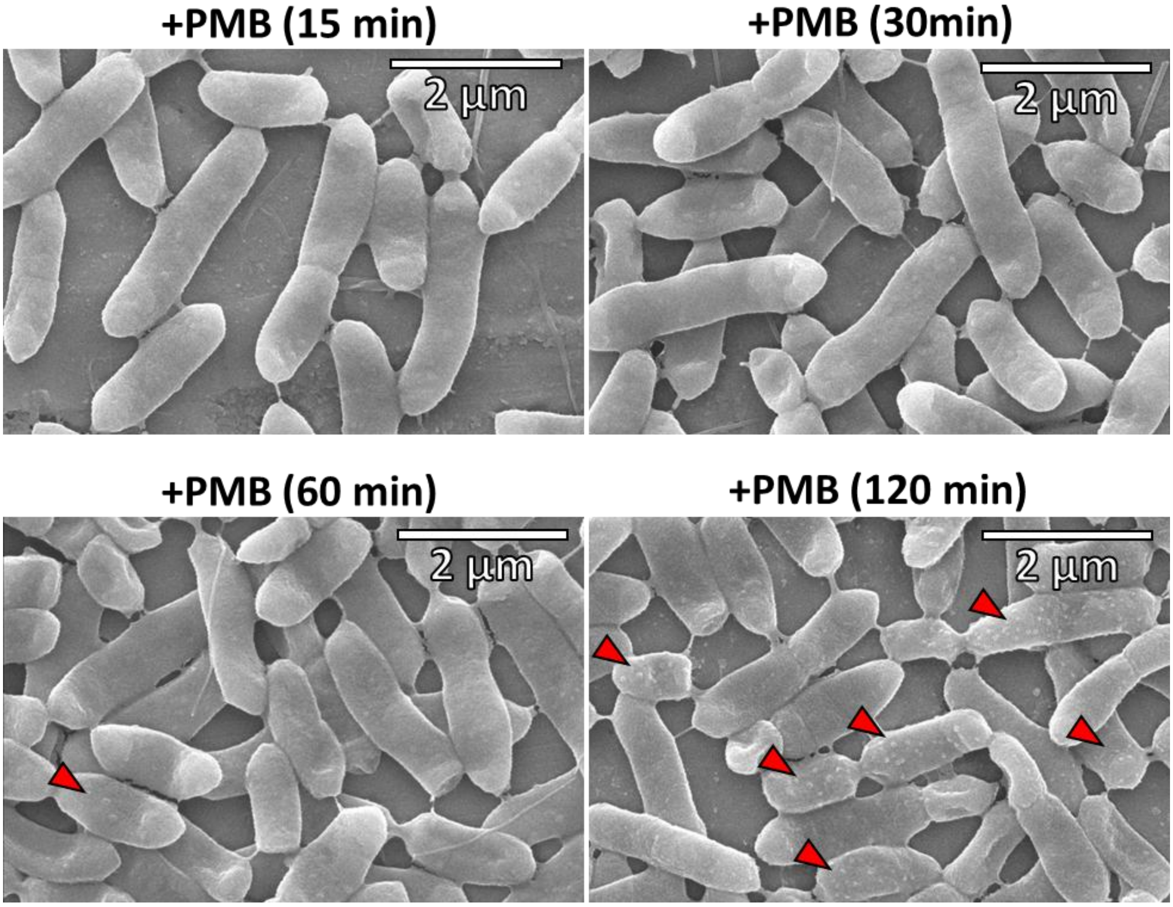

Figure S9. Visualization of PMB binding in *S. detergens* E70 using dansyl-PMB and FM4-64 staining. Confocal laser scanning microscopy (CLSM) was performed to visualize PMB binding to the cell surface of *S. detergens* E70.

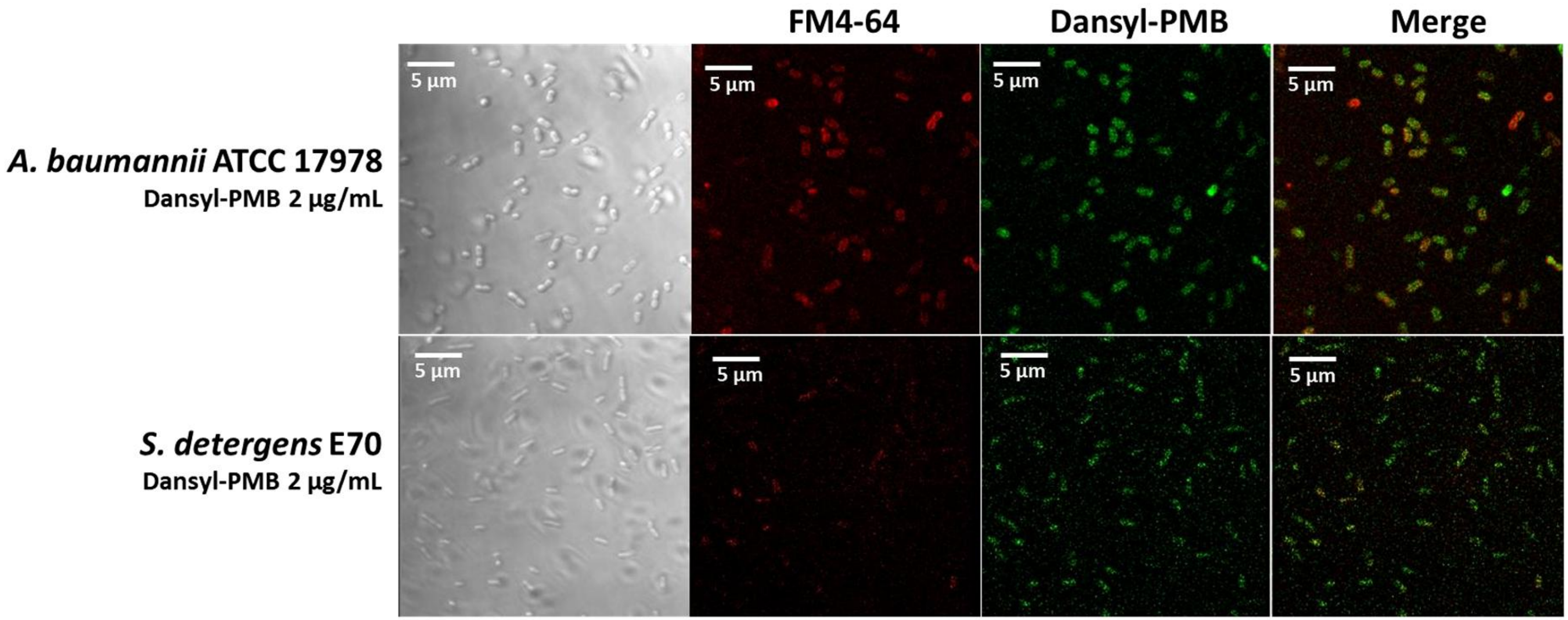

Figure S10. SDS-PAGE analysis of subcellular protein fractions in *S. detergens* E70. SDS-PAGE was performed to analyze proteins isolated from the inner membrane, outer membrane, and periplasmic fractions in the E70 strain. Proteins were separated on 12% polyacrylamide gels and visualized by Coomassie Brilliant Blue staining. All samples were loaded with equal amounts of total protein.

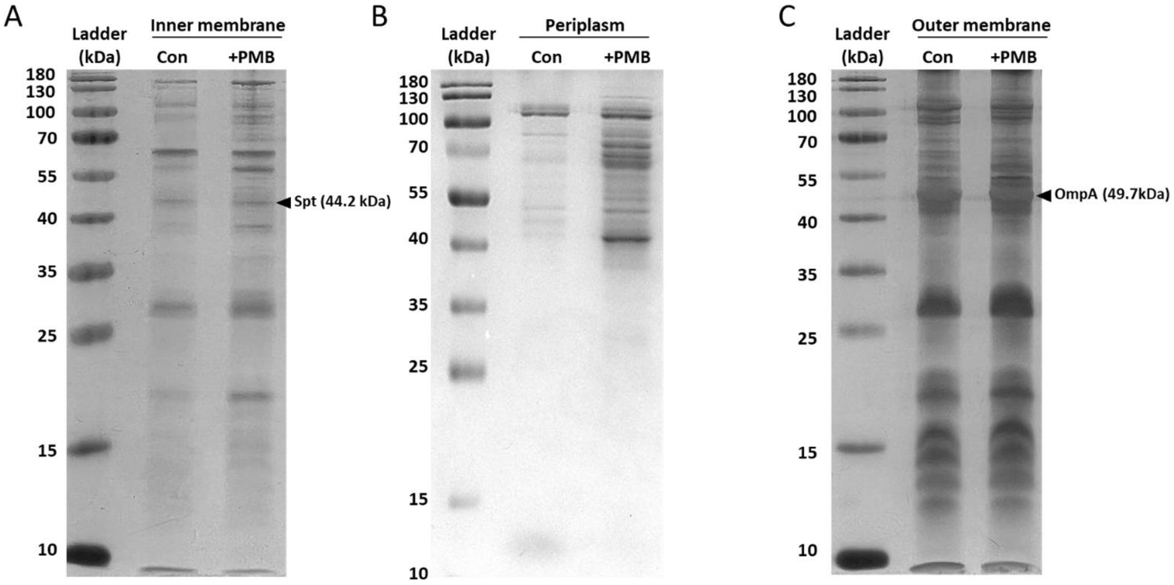

Figure S11. LC-MS and TLC analysis of sphingolipids in *S. detergens* E70 under control and PMB-treated conditions. **(A)** LC-MS chromatograms showing sphingolipid profiles from total lipid extracts of *S. detergens* E70 cultured under control (Con) and ¼ MIC PMB-treated conditions. **(B)** Relative abundances of dihydroceramide and ceramide were quantified based on LC-MS peak areas. **(C, D)** Thin-layer chromatography (TLC) analysis of total lipids showing the Rf 0.35 fractions.

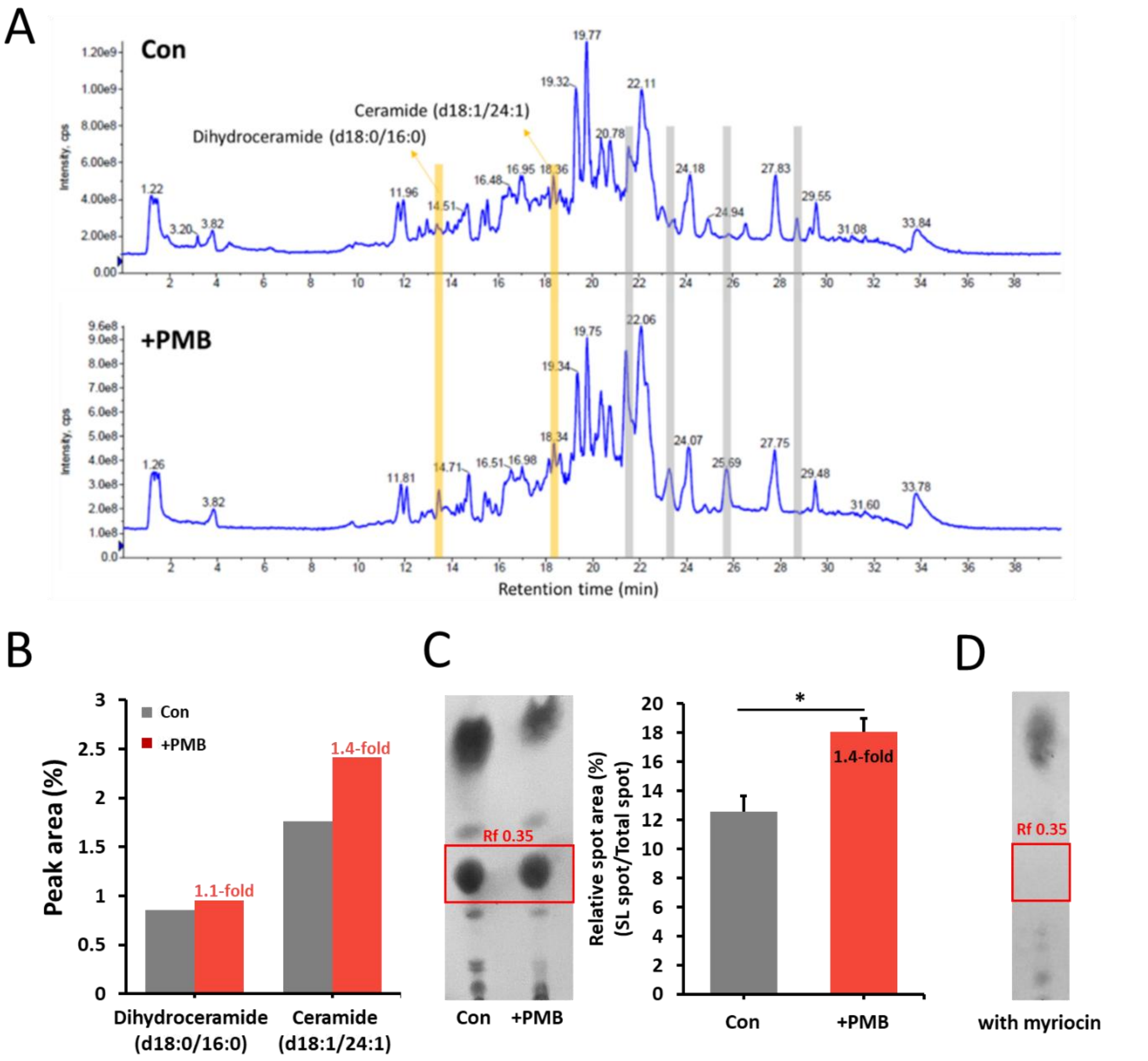

Figure S12. Inhibition of sphingolipid synthesis by myriocin abolishes PMB-induced vesiculation and surface remodeling. (A) FM4-64 fluorescence imaging of OMVs released under PMB treatment (¼ MIC, 64 μg/mL) with or without myriocin (5 μM). These experiments were performed in three independent replicates. (B) Quantification of biofilm biomass by crystal violet staining under control and PMB conditions with or without myriocin. Representative test tube images showing stained biofilms under each condition. These experiments were performed in three independent replicates. (C) CLSM images of FM4-64–labeled cells to assess surface dye incorporation in the presence or absence of myriocin. (D) Zeta potential measurements of cells treated with PMB (¼ MIC, 64 μg/mL) with or without myriocin. (E) Growth rate analysis of cells exposed to PMB (¼ MIC, 64 μg/mL) with or without myriocin. These experiments were performed in three independent replicates. (F) Effect of myriocin on bacterial growth. These experiments were performed in three independent replicates. (*, $p < 0.5$; **, $p < 0.01$; ***, $p < 0.005$; ****, $p < 0.001$; NS, no significant).

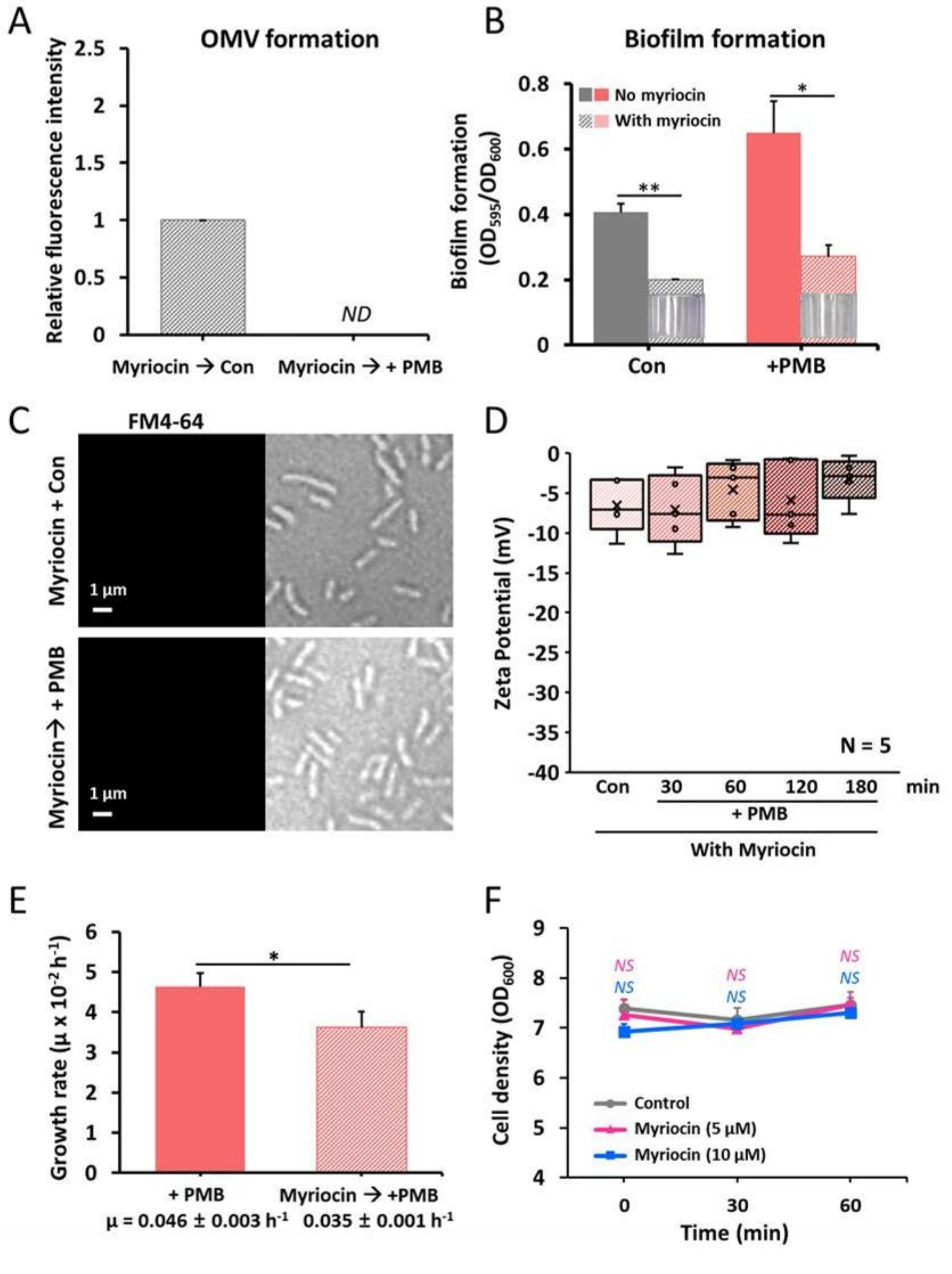
A
OMV formation
Relative fluorescence intensity
2.5
2
1.5
1
0.5
0
ND
Myriocin → Con
Myriocin → + PMB
B
Biofilm formation
No myriocin
With myriocin
Biofilm formation (OD595/OD600)
0.8
0.6
0.4
0.2
0
**
*
Con
+PMB
C
FM4-64
Myriocin + Con
Myriocin → + PMB
1 µm
1 µm
D
Zeta Potential (mV)
0
-5
-10
-15
-20
-25
-30
-35
-40
N = 5
Con
30
60
120
180
min
+ PMB
With Myriocin
E
Growth rate (µ x 10-2 h-1)
6
5
4
3
2
1
0
*
+ PMB
Myriocin → +PMB
µ = 0.046 ± 0.003 h-1
0.035 ± 0.001 h-1
F
Cell density (OD600)
9
8
7
6
5
4
NS
NS
Control
Myriocin (5 µM)
Myriocin (10 µM)
0
30
60
Time (min)

Figure S13 Scanning electron microscopy (SEM) images of *S. detergens* E70 cells after 3 h of exposure to PMB at 1/2, 1/4, and 1/8 of the MIC. Vesiculation and surface protrusions were prominent at higher PMB concentrations but gradually decreased under sub-inhibitory conditions.

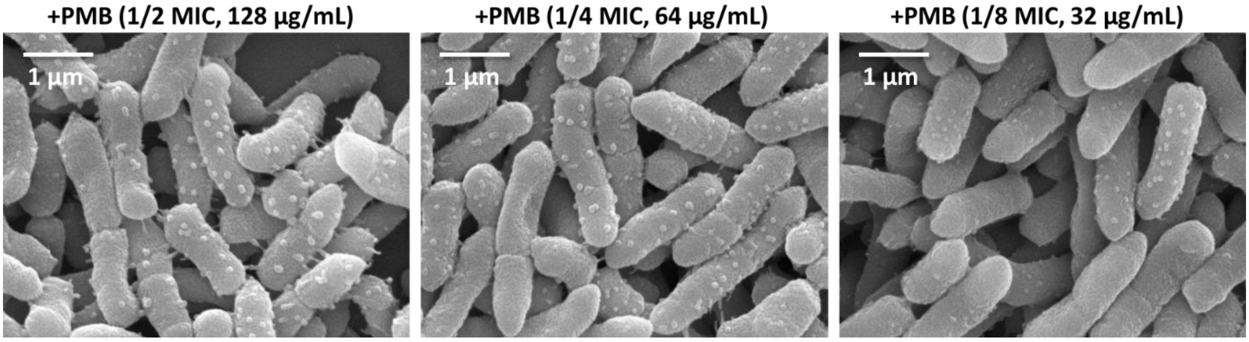

Table S1. Fatty acid composition of *S. detergens* E70 under control and PMB-treated conditions. Relative abundances (%) of individual fatty acid species were determined in *S. detergens* E70 grown under control conditions or treated with ¼ MIC PMB. Fatty acids are grouped into saturated, unsaturated, hydroxy, branched, and summed feature categories. Values represent percentages of total detected fatty acids based on fatty acid methyl ester (FAME) analysis.

Fatty acid	Con	+PMB
**Saturated**		
C9:0	-	0.53
C11:0	0.53	-
C12:1	0.16	-
C14:0	2.38	1.77
C16:0	11.07	8.3
C18:0	0.72	0.82
**Unsaturated**		
C14:1 w5c	0.84	0.83
C15:1 w6c	-	0.3
C16:1 w5c	-	0.3
C17:1 anteiso w9c	-	0.68
**Hydroxy**		
C8:0 3OH	-	1.3
C10:0 2OH	0.29	-
C14:0 2OH	0.51	0.42
C16:0 2OH	0.28	0.19
C16:0 3OH	2.92	2.84
iso-C11:0 3OH	0.51	0.59
iso-C15:0 3OH	4.39	4.38
iso-C17:0 3OH	6.42	8.62
**Branched**		
iso-C10:0	1.62	-
iso-C13:0	0.28	-
iso-C15:0	24.27	23.61
C15:0 anteiso	0.5	0.6
C17:0 anteiso	0.21	0.24
**Summed feature**		
2	0.23	0.34
3	41.02	42.29
9	0.83	1.06

Table S2. List of primers used in this study.

Primer	5'-3' sequence	Reference
*spt*_RT_F	GTGGATTCGTAGCGGGTGAT	This study
*spt*_RT_R	ACGCCTTCAGTGTAGAAGCAA	This study
*eptA*_RT_F	TCTGCTCTTTTAATCCCGCCA	This study
*eptA*_RT_R	ACCTCCGGTCCACATCAAGG	This study
